# Effects of interventions with resistance exercises on muscle strength, physical disability, and quality of life in systemic sclerosis patients: a systematic review with meta-analysis

**DOI:** 10.1186/s42358-025-00468-1

**Published:** 2025-07-28

**Authors:** André Luiz Silveira Mallmann, Daniel Nóbrega de Moraes, Lucas Denardi Dória, Leonardo Peterson dos Santos, Stephanie Pilotti, Mayra Angélica de Souza Antunes, Laura Fontana Steinmetz, Thauan Júnior Santos de Souza, Vanessa Hax, Jerri Luiz Ribeiro, Ricardo Machado Xavier, Rafael Mendonça da Silva Chakr

**Affiliations:** 1 Postgraduate Program in Medical Sciences, Universidade Federal do Rio Grande do Sul, Porto Alegre, Rio Grande do Sul Brazil; 2 Hospital de Clínicas de Porto Alegre, Porto Alegre, Rio Grande do Sul Brazil; 3 Postgraduate Program in Human Movement Sciences, Universidade Federal do Rio Grande do Sul, Porto Alegre, Brazil; 4 Hospital de Clínicas de Porto Alegre, 2350, Ramiro Barcelos St., Porto Alegre, RS 90420-010 Brazil

**Keywords:** Scleroderma, Muscle strength, Physical disability, Quality of life, Resistance training, Physical rehabilitation, Occupational therapy

## Abstract

**Introduction:**

Systemic sclerosis (SSc) often leads to decreased muscle strength and mass, impairing physical performance and causing disability. Interventions with resistance exercise (RE) is an effective non-pharmacological approach to mitigate these issues. This systematic review aims to evaluate the effects of interventions with RE on muscle strength, muscle mass, physical performance, physical disability, and quality of life (QOL) in SSc patients, as well as to assess its adherence and safety.

**Methods:**

A systematic review and meta-analysis were conducted based on a PICOS framework: Patient = Systemic Sclerosis; Intervention = Resistance exercise; Study design = Randomized clinical trials. Searches were performed across MEDLINE (PubMed), PMC, Web of Science, Cochrane Library, LILACS, and EMBASE up to January 2025.

**Results:**

Ten randomized clinical trials, including 422 participants (~85% female), were eligible for analysis. Participants’ ages ranged from 42 to 64 years, with body mass indices between 22.5 and 28.0 kg/m^2^. The intervention period was standardized to 12 weeks. Interventions with RE significantly improved muscle strength (SMD = 2.76 kg; 95% CI, 1.32 to 4.20; *p* = 0.0002) and functional disability (SMD = −0.47; 95% CI, −0.93 to −0.00; *p* = 0.05) compared to controls. Interventions with RE also showed superiority in the physical component of QOL (SMD = 0.42; 95% CI, 0.04 to 0.81; *p* = 0.03). Although enhanced physical performance was observed, statistical pooling was not possible due to limited data. Interventions with RE had a low incidence of adverse events, but data on disease progression and adherence were insufficient.

**Conclusion:**

Interventions with RE benefits muscle strength, physical function, and QOL in SSc patients, though optimal protocols and adherence strategies need further investigation. More robust studies are required to refine training methods and enhance clinical trial designs.

**Supplementary Information:**

The online version contains supplementary material available at 10.1186/s42358-025-00468-1.

## Introduction

Systemic sclerosis (SSc) is a rare auto-inflammatory rheumatic disease characterized by connective tissue and multiorgan fibrotic manifestations, coupled with the presence of vasculopathy [1]. SSc patients frequently experience a notable reduction in muscle strength and mass, which can result in physical disability and a diminished quality of life [2–5]. This deterioration in muscle function is linked to an intensification of clinical manifestations and may accelerate the progression of the disease [6, 7]. These muscular changes have a detrimental impact on the overall health and quality of life of SSc patients, underscoring the necessity for early detection and management strategies as the interventions with resistance exercises that has proven effective in other populations [8–11]

RE commonly refers to movements performed against resistance that may not necessarily follow a periodized structure, such as exercises using elastic bands or rehabilitation/physiotherapy squeeze balls for the hands [12, 13], whereas resistance training (RT), also known as strength training, is defined as a structured and periodized program of resistance exercises designed with specific goals in mind [14–16]. The European League Against Rheumatism (EULAR) [17] has recommended RE as part of a non-pharmacological management plan for SSc, highlighting its potential to improve hand function, reduce physical disability, and enhance health-related quality of life. A systematic review, from 2019 [18], included 9 studies and summarized the effects of exercise therapy on physical functioning in SSc patients. They found improvements of hand function, mouth opening, aerobic capacity and/or muscle strength, considering all kinds of exercise. Another systematic review, from 2024 [12], investigated the efficacy of lifestyle interventions for the management of SSc, with a total of 36 studies included, and concluded that physical exercise and patient education should be considered for improving physical function in SSc patients. Considering the beneficial characteristics linked to RE as an adjunctive therapeutic modality in the management of SSc, it is noteworthy that, to date, no comprehensive systematic reviews have been conducted to elucidate the impact of RE as an intervention on muscle strength, muscle mass, physical performance, physical disability and quality of life (QOL) in SSc patients.

Therefore, this systematic review aims to summarize the current evidence on the effects of RE on muscle strength, muscle mass, physical performance, physical disability, and QOL in SSc patients. Also, to verify the levels of adherence and safety of RE for SSc patients.

## Methods

### Registration

This systematic review with meta-analysis was conducted following the guidelines of the PRISMA statement (check-list presented at the Supplemental Digital Content 1) [19]. This study has been registered in PROSPERO under the registration number CRD42022368918.

### PICOS format

This systematic review with meta-analysis was based on a focused question outlined in a PICOS format [20]: Patient/Problem/Population = Systemic Sclerosis; Intervention = Resistance exercises; and Study design = Randomized clinical trials.

#### Data sources

The electronic databases used were MEDLINE (PubMed), PMC, Web of Science, Cochrane Library, LILACS, and EMBASE up to January 2025. We employed a comprehensive search strategy tailored to the specifics of each database. Additionally, we examined the references of the included studies and searched the “gray” literature for studies that aligned with our PICOS strategy. In cases where data were missing, we reached out to the authors of the selected studies.

#### Search terms

We used keywords and medical subject headings (MeSH) for terms like “systemic sclerosis” and “resistance training” or “rehabilitation” or “occupational therapy”. No filters were applied to this search. The complete string used for PubMed is fully outlined in Supplemental Digital Content 2

#### Inclusion/Exclusion Criteria

The inclusion criteria were: (1) Participants diagnosed with SSc according to ACR/EULAR criteria [21], (2) Randomised clinical trials that allowed comparison of resistance exercise - which we considered to be any type of exercise that involves voluntary skeletal muscle contraction against some resistance (e.g. elastic bands, squeeze balls, barbells, dumbbells, resistance training machines, etc), with or without a well-structured or periodized program - with other training interventions or with a control group (no intervention or placebo training) (CON), (3) studies that assessed at least one of the following outcomes: muscle strength, muscle mass, physical performance, physical disability or quality of life, and (4) studies that provided pre- and post-intervention data. We considered protocols with a well-structured or periodized exercise intervention as RT while those in with the intervention was not well structured or described as RE. There were no restrictions on the publication date. The exclusion criteria were (1) manuscripts not written in English, Portuguese, or Spanish, (2) reviews, meta-analyses, observational, quasi-experimental studies, or conference studies.

### Study selection and data extraction

Title, abstract, and full text screening was performed in pairs by four independent reviewers (Antunes, MAS, Steinmetz, LF, Doria, LD, and Pilotti, S). The reviewers extracted data from studies independently and disagreements were resolved by discussion.

If there was conflict, a third and fourth reviewers (Mallmann, ALS) provided the final decision.

All data from each study were screened using a bibliographic management site (rayyan.ai) and a citation manager (Mendeley, version 1.17.9).

The data extracted included (1) population characteristics, (2) outcome measures, (3) methods, (4) exercise/interventional characteristics, and (5) the main study result. If intervention effects were assessed at multiple time points, we considered, for the meta-analyses, until 12 weeks. When available, data were extracted in the form of delta mean (mean change), delta standard deviation (SD change), and sample size. In instances of incomplete raw data availability, we attempted to contact the corresponding author or extrapolated data from figures. For cases where studies reported baseline and post-intervention outcomes without mean change and SD change, we calculated the delta value using the equation (Delta mean = post-training mean - baseline mean). Cochrane’s handbook [22] provides a correlation estimation formula considering one of included studies in which data are presented as mean ± standard deviation to calculate the SD change. However, as none of the included studies provided data as mean ± standard deviation, we considered a correlation of zero, following the recommendations of the Cochrane’s handbook [22]. The following formula was used to find SD change for selected studies: [formula to be provided based on Cochrane’s handbook guidelines].

1 $$\eqalign{& S{D_{E,change}} = \cr & \sqrt {SD_{E,baseline}^2 + SD_{E,final}^2{\mkern 1mu} - \left( \matrix{2{\mkern 1mu} \times {\mkern 1mu} Corr{\mkern 1mu} \times \hfill \cr S{D_{E,baseline}} \times S{D_{E,final}} \hfill \cr} \right)} \cr} $$

Where Corr is correlation coefficient in the experimental group, SD_E,baseline_ is baseline standard deviation in the experimental group, SD_E,final_ is final standard deviation in the experimental group and SD_E,change_ is standard deviation of the changes in the experimental group. When data were presented by interquartile range (IQR), it was decided to transform these data in order to standardize the results of all studies in mean change and SD_change_. The equation used to calculate the mean change is available below.

2 $$x\, \approx \frac{{{q_1} + m\, + \,{q_3}}}{3}$$

Where q1 is the first quartile, m is the median and q3 is the third quartile. Finally, to find the SD_change_ presented by IQR, we use the calculation available below.

3 $$\,S\, \approx \,\frac{{{q_3}\, - \,{q_1}}}{{1.35}}$$

The choice for using these formulas was based on a previous systematic review with meta-analysis about effects of blood flow restriction training (BFR) training on muscle strength, hypertrophy, and functionality for people with osteoarthritis and RA [11].

### Methodological quality assessment

The Physiotherapy Evidence Database (PEDro) scale was used to assess methodological quality for each of included studies. Two independent reviewers performed the evaluation of methodological quality (Antunes, MAS and Steinmetz, LF). In cases of disagreements, a third reviewer (Mallmann, ALS) performed the final decisions. PEDro scale is composed of: external validity (item 1), internal validity (items 2 - 9), and statistical reports (items 10 - 11) [23]. The maximum score was 10 points and t scores < 4 points was considered as “bad”, 4 – 5 was considered as “regular”, 6 – 8 was considered as “good”, and 9 – 10 was considered “excellent” [24, 25]. Studies were included independently of the methodological quality calculated.

### Risk of bias

The risk of bias of the studies was assessed using the risk of bias tool 2.0 (RoB2) from Cochrane to randomized clinical trials [26]. Two authors (Moraes, DN and Dos Santos, LP) independently assessed the risk of bias. When necessary, a third reviewer performed the final decision (Mallmann, ALS). The evaluators analyzed the randomization process, deviations from intended interventions, missing outcome data, measurement of the outcome, and selection of the reported results. The studies were classified into low, moderate, or high risk of bias.

### Evidence quality assessment

Evidence quality was determined using the Grading of Recommendations Assessment, Development and Evaluation (GRADE), by the GRADEpro Guideline Development tool [software] [27]. For each of the 7 items of the GRADE scale, two reviewers (Moraes, DN and Dos Santos, LP) assessed the studies independently. A third reviewer (Mallmann, ALS) resolved disagreements when necessary. The GRADE approach considers the risk of bias and the body of evidence to rate the certainty of the evidence into one of four levels: high certainty, moderate certainty, low certainty, and very low certainty [28].

#### Statistical analyses

We conducted the meta-analysis using mean_change_ and SD_change_ from each study. All outcome measures were continuous variables. Two meta-analyses, representing the effects of interventions, were performed: the random-effects model with the mean difference (MD) or standardized mean difference (SMD). MD was performed when studies reported outcomes with the same assessment scale or instrument. When the same outcomes between studies were evaluated but analyzed by different scales or instruments, we performed SMD [11]. The calculation of SMD is represented by dividing the difference in mean outcome between groups by the standard deviation of the result within the groups. The formula between groups within each study used is available below.

4 $${S_{within}} = \sqrt {\frac{{\left( {{n_{1\,}} - 1} \right)S_1^2\, + \left( {{n_2}\, - 1} \right)S_2^2}}{{{n_1} + \,{n_{2\,}} - 2}}} $$

The 95% confidence intervals (CI) were used, and the heterogeneity of the studies included in the meta-analysis was assessed using the inconsistency test (I^2^). Inconsistency was considered as low, moderate or high when values were 25%, 50% and 75% or more respectively [20, 26]. The software used for statistical analysis was RevMan (Review Manager 5.4.1, The Cochrane Collaboration, 2020), and we considered it statistically significant when ´*p* < 0.05.

## Results

### Literature search

**Fig. 1:**
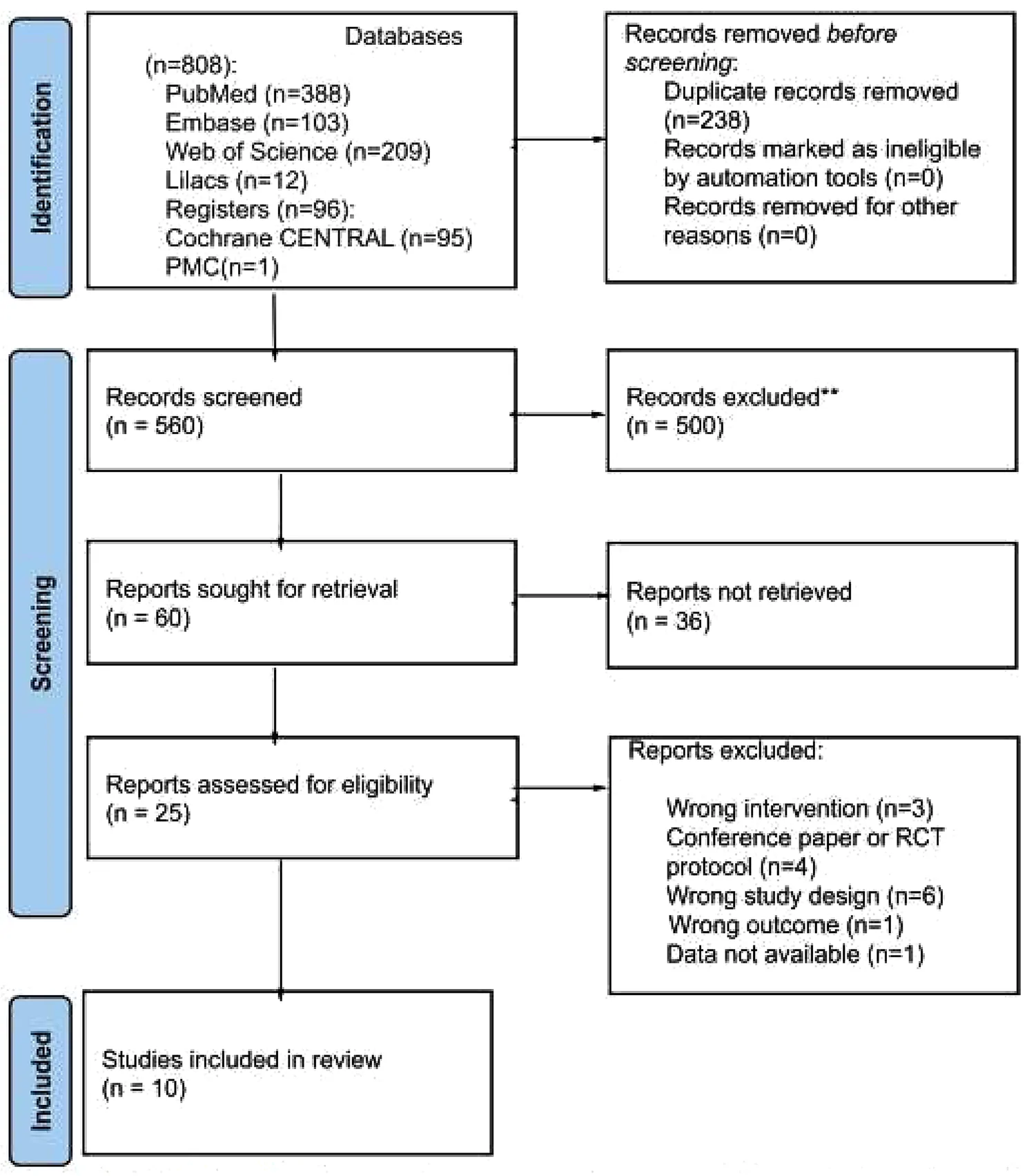
PRISMA 2020 flow diagram for new systematic reviews which included searches of databases, registers and other sources

We identified 808 potentially relevant full studies (238 duplicate publications) based on the search strategy described at the initial search stage. After title, abstract, and full-text screening, 561 studies were excluded in accordance with inclusion/exclusion criteria. Therefore, 10 [29–38] relevant full studies were included in the review. Fig. 1, in accordance with the new PRISMA recommendations [39], shows the flow diagram of study selection.

The flowchart is presented at Fig. 1

### Sample characteristics

Among the 10 included studies, a total of 422 (~85% female) participants were enrolled, with mean ages varying from 41.96 to 63.60 years old and body mass index from 22.46 to 27.72 kg/m^2^. More details are presented in Table 1 Most training [31–36] was based on wrists and fingers stretching/mobility associated with resistance training for flexion/extension of wrists and fingers (e. g. elastic bands resistance). There was a wide range of RE among included studies with most of them focusing on hand rehabilitation, which may be considered very important to SSc patients. In the majority of studies, the intervention group was compared with a control group. Only two included studies compared RE against another intervention. Yakut et al. (2021) [30] compared RI combined with aerobic training (AT) and breathing exercises vs home-based breathing exercises, combined with posture exercises and walking, both without supervision. Piga et al. (2014) [33] compared an intervention with a device, developed and provided by their group, vs the same exercises performed with common objects, both under minimal remote supervision by physicians. At Table 2 we presented a short description of each protocol for the included studies and the outcomes extracted from each included study. Also, we separate studies in two categories, related to its training characteristics: resistance training (*n* = 4) [29, 30, 37, 38]when the protocol involved clear structures, goals and/or training progression; and resistance exercise (*n* = 6) [31–36]: when there were no clear informations about protocols, structure and/or progression of exercises. The complete characterization of frequency, sets, repetitions, intensity, and interval protocols for included studies are available at the supplemental digital content 3

**Table 1:** Characteristics of participants for each of included studies

Authors (Year)	Group	Age (years)	p	BMI (kg/cm^2^)	p	Disease duration (years)	p	mRSS	p	Limited, n (%)	Diffuse, n (%)	p	Female, n (%)	Male, n (%)	p	Raynaud’s Phenomenon n (%)	p	Pulmonary arterial hypertension n (%)	p	Digital ulcers n (%)	p	Corticoid use (%)
**Schouffoer et al. (2011) [**37**]**	IG	53.9 ± 10.8	0.46		NI	6.5 (8.2)	0.71	5.0 ± 4.0	0.86	15 (53.6)	13 (46.4)		19 (67.9)	9 (32.1)	0.29	8.6 (12.7)	0.69	5 (17.9)	0.43	NI	NI	Y (14)
	CG	51.7 ± 10.8				8.2 (10.5)		5.2 ± 6.2		15 (60.0)	10 (40.0)		21 (84.0)	4 (16)		10.3 (12.9)		2 (8.0)	NI			Y (4)
**Piga et al. (2014) [**33**]**	IG	57.0 ± 10.0	NI	NI	NI	6.9 ± 4.1	NI	NI		8 (80)	2 (20)	NI	10 (100)	NA		NI		NI		NI		NI
	CG	57.4 ± 11.7		NI		6.7 ± 4.2		NI		8 (80)	2 (20)		10 (100)	NA		NI				NI		NI
**Horváth et al. (2017)** [32]	IG	59.7 (14.5)	0.54	NI	NI	11.1 (6.7)	0.34	NI		NI	NI		29 (93)	2 (7)	0.720	NI		16 (52%)	0.23	2 (7%)	0.18	NI
	CG	62.1 (8.4)		NI		13.0 (7.6)		NI		NI	NI		20 (91)	2 (7)		NI		15 (68%)		4 (18%)		NI
**Filippetti et al. (2020) [**29**]**	IG	63.60 ± 10.40	N/S	26.20 (24.00–28.30)	NI	10.00 (6.00–16.00)	NI	8.60 ± 5.50	NI	(56.00)	(44)	N/S	(80.00)	(20.00)	N	NI	NI	NI	NI	NI	NI	Y (16.7)
	CG	61.80 ± 14.40		24.70 (23.50–28.20)		11.50 (7.00–17.50)		6.50 ± 3.90		(75.00)	(25)		(79.20)	(21.80)		NI		NI		NI		Y (12)
**Mitropoulos et al. (2020)** [33]	IG	69.6 ± 11.4	NI	24.8 ± 3.1	NI	8 ± 2	NI		NI	NI	NI	NI	NI	NI	NI	NI	NI	NI	NI	NI	NI	NI
	CG	63.6 ± 12.2		26.6 ± 4.6		8 ± 2			NI		NI	NI		NI	NI		NI		NI		NI	
**Yakut et al. (2021) [**30**]**	IG	51.21 ± 11.46	0.62	24.85 ± 3.20	0.207	9.71 ± 4.93	0.25	9.30 (3.0–20.0)	0.68	15 (79)	4 (21)	0.66	16 (84)	3 (16)	0.94	17 (90)	0.66	10 (53)	0.67	5 (26)	0.69	NI
	CG	49.55 ± 8.14		26.31 ± 5.59		8.61 ± 5.94		9.10 (3.0–19.0)		16 (89)	2 (11)		15 (83)	3 (17)		15 (83)		11 (61)		3 (17)		NI
**Roque et al. (2022)** [34]	IG	44.08 ± 10.60	0.21			91.30 ± 76.89 (months)	0.64			9 (75)	3 (25)	1.00	12 (100)	0 (0)	0.47	11 (100)	0.21	1 (9)	1.00	4 (36)	0.37	Y (36)
	CG	50.75 ± 11.08				123.82 ± 100.55 (months)				9 (75)	3 (25)		10 (83)	2 (17)		9 (75)		1 (8)		2 (16)		Y (8)
**Gokcen et al. (2022) [**31**]**	IG	54.5 (47.0 - 63.5)	0.89	25.8 (22.9–29.3)	0.972	11.0 (5.5–23.0)	0.71	10.0 (6.0–13.5)		25 (100)		0.89	23 (71.9)		0.546			NI	NI	NI	NI	NI
	CG	56.0 (50.0 - 61.0)		25.9 (22.9–29.0)		9.0 (5.8–21.3)		5.0 (4.0–13.0)		9 (30.0)	21 (70.0)		25 (100)			21 (70.0)		NI		NI		NI
**Sahin et al. (2023) [**35**]**	IG	53.43 ± 11.95	0.22	27.72 ± 2.98	0.488	6.04 ± 3.19	0.23	NI		21 (91.3)	2 (8.7)		NI	NI	NI	NI	NI	NI	NI	NI	NI	NI
	CG	57.60 ± 10.97		27.04 ± 3.53		7.60 ± 5.20		NI		21 (91.3)	2 (8.7)		NI	NI		NI		NI		NI		NI
**Sari et al. (2023) [**36**]**	IG	42.46 ± 14.99	NI	23.55 ± 5.86	NI	7.75 ± 6.81	NI	NI	NI	8 (33.3)	16 (66.7)	NI	23	1	NI	0.13 (0–0.38)	NI	NI	NI	0	NI	4.1
	CG	41.96 ± 10.62		22.46 ± 4.54		8.13 ± 7.50		NI		7 (31.8)	15 (68.2)		22	0		0.20 (0.06–0.33)		NI		1		18

IG: Intervention group; CG: Control group; BMI: Body mass index; mRSS: Modified Rodnan skin score; Y: yes; n: Number of participants; -: not informed; *: data presented in months from the beginning of Raynaud’s phenomenon

**Table 2:** Characteristics of interventions and pre- vs post-interventions outcomes

				Intervention		Comparator		Control	
	Authors (Year)	Intervention/comparator/control description	Outcome measure	n	Delta ± SD	n	Delta ± SD	n	Delta ± SD
**Resistance exercise**	Piga et al. (2014) [33]	IG: Hand kynesiotherapy exercises with a portable device, It consisted of 4 strengthening and 3 mobility exercises. CG: Hand kynesiotherapy exercises with common daily use objects, It consisted of 3 strengthening and 3 mobility exercises,	Handgrip (mmHg) R	10	15.9 ± 36.82	10	15.00 ± 47.29	-	NA
			Handgrip (mmHg) L	10	15.3 ± 44.3	10	11.60 ± 43.28	-	NA
			Handpinch (mmHg) R	10	19.70 ± 37.74	10	21.30 ± 16.80	-	NA
			Handpinch (mmHg) L	10	19.40 ± 38.05	10	13.10 ± 28.66	-	NA
			HAQ	10	−0.68 ± 0.72	10	−0.47 ± 0.86	-	NA
			SF-36 Mental	10	1.20 ± 19.50	10	−4.60 ± 21.13	-	NA
			SF-36 Physical	10	0.70 ± 14.79	10	3.50 ± 16.40	-	NA
	Horváth et al. (2017) [32]	IG: complex physiotherapy program including thermal baths medical massages of upper extremities exercise (30 minutes/day, isometric, isotonic and stretching hand exercises; ergotherapy CG: The control group received similar therapy to their large joints, and the spine without being a subject to any treatment to the hands	Isometric handgrip strength (kg) L	31	2.12 ± 10.13	-	NA	22	−0.05 ± 12.68
			Isometric handgrip strength (kg) R	31	−0.57 ± 9.83	-	NA	22	0.64 ± 11.16
			HAQ-DI	31	−0.13 ± 1.05	-	NA	22	0.30 ± 0.59
			SF-36 Mental	31	0.40 ± 21.20	-	NA	22	−3.33 ± 19.45
			SF-36 Physical	31	1.80 ± 18.41	-	NA	22	3.17 ± 14.55
	Gokcen et al. (2022) [31]	IG: 1-hand exercise training (isometric exercise with an exercise ball and self-administered stretching) with supervision CG: Education about SSc + booklet with recomedations	Isometric handgrip strength (kg)	32	5.50 ± 8.65*	-	NA	30	−0.33 ± 12.61
			HAQ-DI	32	−8.00 ± 8.61*	-	NA	30	0.67 ± 23.05
			SF-36 Mental	32	−0.67 ± 28.46	-	NA	30	−12.80 ± 51.32
			SF-36 Physical	32	−9.83 ± 35.38*	-	NA	30	−5.83 ± 58.94
	Roque et al. (2022) [34]	IG: Free active and resisted active exercises (fingers, fist and hand grip strength) for both hands CG: Booklet with information about the disease	Isometric handgrip strength (kg)	12	6.24 ± 7.74	-	NA	12	1.79 ± 12.31
			SHAQ	12	−0.13 ± 0.86	-	NA	12	−0.05 ± 0.87
			SF-12 Mental	12	6.21 ± 18.09	-	NA	12	3.98 ± 17.65
			SF-12 Physical	12	2.70 ± 11.54	-	NA	12	−2.89 ± 13.54
	Sahin et al. (2023) [35]	IG: Upper extremity home exercises (stretching and strengthening) CG: Patient education	Muscle Strength L	23	2.79 ± 11.26*	-	NA	23	−0.36 ± 14.83
			Muscle Strength R	23	2.32 ± 11.82*	-	NA	23	−0.58 ± 12.86
			COPM: Canadian Occupational Performance Measure	23	1.09 ± 2.05*	-	NA	23	−0.31 ± 2.25
			DASH	23	0.00 ± 0.00*	-	NA	23	0.00 ± 0.00
	Sari et al (2023) [36]	IG: “Clinical Pilates-based exercises were given to the Telerehabilitation-based treatment. CG: Orientation: Electronic supplementary material. The exercises given to the patientsin a home program were the same asthose in the telerehabilitation-based exercise program.	NHP	24	−162.57 ± 24.00*	22	−60.00 ± 72.82	-	NA
**Resistance Training**	Schouffoer et al. (2011) [37]	IG: General exercises (AT + RT), hand/mouth exercises, and educational sessions CG: Usual outpatient care	Isometric handgrip strength (kg)	28	2.20 ± 2.37*	-	NA	25	−0.40 ± 3.48
			6-minute walk distance (m)	28	43.83 ± 32.96*	-	NA	25	3.90 ± 36.52
			HAQ-DI	28	−0.18 ± 0.26*	-	NA	25	0.13 ± 0.21
			SF-36 Mental	28	0.00 ± 4.30	-	NA	25	0.57 ± 5.56
			SF-36 Physical	28	2.10 ± 5.33	-	NA	25	−0.50 ± 5.78
	Filipetti et al. (2019) [29]	IG: Home-based combined exercise training (RT + AT) without supervision CG: Phone calls to evaluate the health status	Isometric handgrip strength (kg)	22	1.33 ± 9.27*	-	NA	22	−0.30 ± 8.07
			Quadriceps strength (kg)	22	4.10 ± 7.81*	-	NA	22	−0.60 ± 7.02
			Biceps strength (kg)	22	2.30 ± 4.72*	-	NA	22	−0.73 ± 4.09
			Fat free mass (kg)	22	0.30 ± 6.60*	-	NA	22	−0.13 ± 6.13
			6-minute walk distance (m)	22	32.33 ± 56.07*	-	NA	22	−3.33 ± 64.60
			HAQ-DI	22	−0.12 ± 0.34*	-	NA	22	0.00 ± 0.35
			SF-36 Mental	22	2.50 ± 6.29	-	NA	22	−0.83 ± 6.77
			SF-36 Physical	22	5.53 ± 6.77*	-	NA	22	−1.20 ± 7.86
	Mitropoulos et al. (2020) [33]	IG: Combined exercise training (HIIT + RT) under supervision. CG: “Control group did not perform the exercise programme.	EQ-5D-5 L - Usual activities	16	−0.19 ± 1.06	-	NA	16	0.45 ± 1.41
			EQ-5D-5 L - Anxiety	16	−0.32 ± 0.67	-	NA	16	0.11 ± 1.39
	Yakut et al. (2021) [30]	IG: Combined exercise training (breathing + RT + AT) under supervisionCG: Home-based Breathing + posture + walking without supervision	Isometric handgrip strength (kg)	20	3.74 ± 8.28*	20	2.12 ± 8.37	-	NA
			Dominant side knee extensors (kg)	20	4.61 ± 2.55*	20	0.60 ± 2.78	-	NA
			6-minute walk distance (m)	20	142.57 ± 100.21*	20	48.26 ± 98.26	-	NA
			SHAQ-global	20	−0.39 ± 0.19*	20	−0.05 ± 0.07	-	NA
			SF-36 Mental	20	34.39 ± 18.49*	20	3.25 ± 21.41	-	NA
			SF-36 Physical	20	34.07 ± 19.02*	20	5.03 ± 23.71	-	NA

*: indicates statistical significance provided by the authors; IG: Intervention group; CG: Comparator or control group; Control: Control group; RT: Resistance training; AT: Aerobic training; HAQ-DI: Health Assessment Questionnaire Disability Index; SF-36: Short Form Health Survey; SF-12: Short Form Health Survey; SHAQ: Scleroderma Health Assessment Questionnaire; COPM: Canadian Occupational Performance Measure; DASH: Disabilities of the Arm, Shoulder and Hand; EQ-5D-5 L: Questionnaire for health assessment; NA: Not Applicable

### Muscle strength

**Fig. 2:**
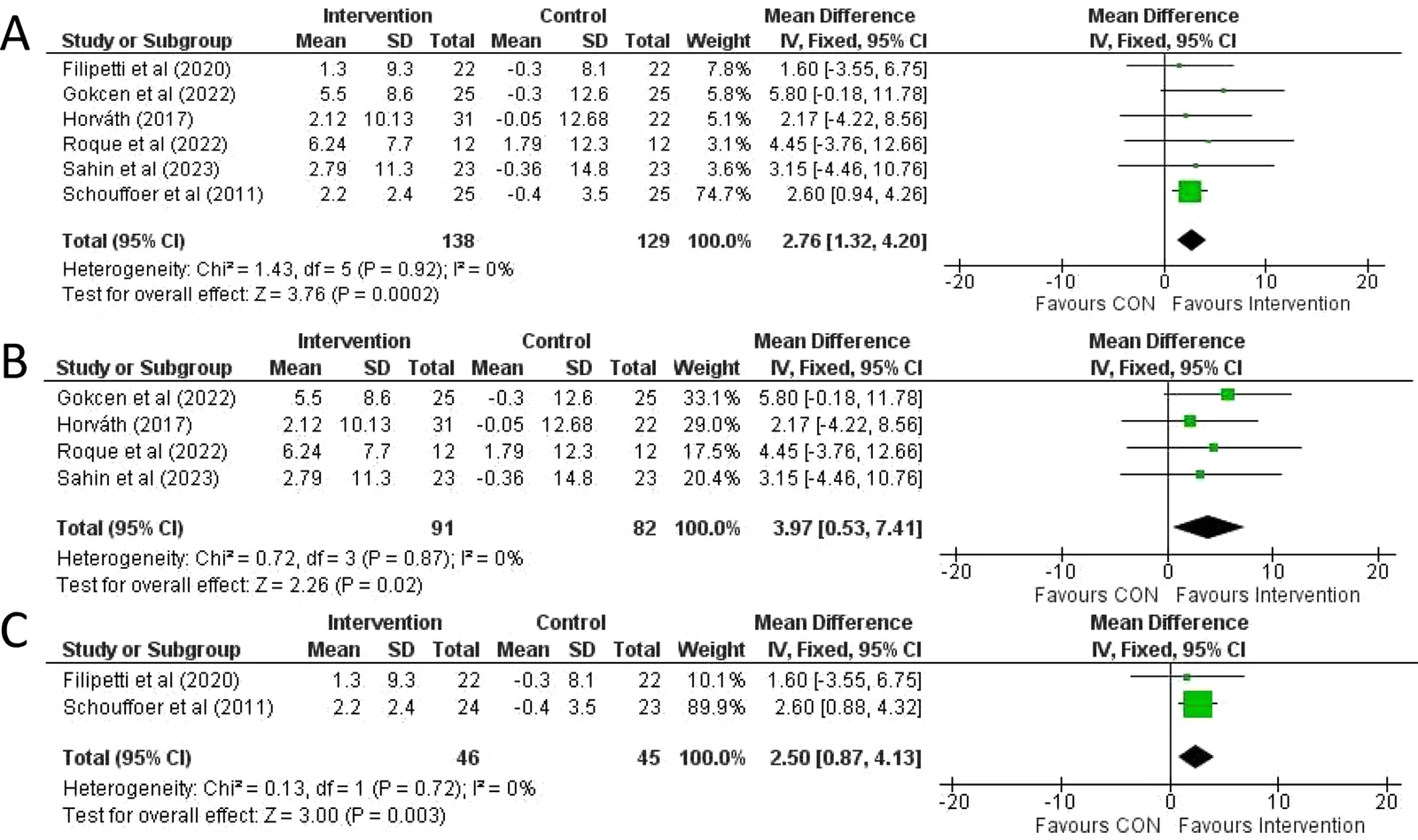
Effects of interventions on muscle strength assessed by isometric handgrip strength (kilograms). **A**. Analysis with all studies that evaluated strength by isometric handgrip dynamometer (*n* = 5). **B**. Sensitivity analysis taking off studies that involved resistance training. **C**. Sensitivity analysis taking off studies that involved resistance exercises. Abbreviations: CON: control; I2: inconsistency of studies; SD: standard deviation; SMD: standardized mean difference; 95% CI: 95% confidence interval; IV: inverse variance; fixed: fixed effects model

Eight of the included studies evaluated muscle strength by isometric handgrip test [29–35, 37]. Three studies included different outcome measures for muscle strength. Yakut et al. (2021) evaluated by dominant knee extensor isometric dynamometry. Filipetti et al. (2019) chose biceps (forearm flexion) and quadriceps (leg extension) isometric dynamometry added to isometric handgrip strength. Piga et al. (2014) evaluated handpinch isometric strength dynamometry. From the ten included studies, six had data extracted for meta-analyses comparing RE and CON on muscle strength in SSc patients [29, 31, 32, 34, 35, 37] (Fig. 2). The mean difference was 2.76 kg (95% CI, 1.32 to 4.26, I^2^ = 0%; *p* = 0.0002) on handgrip in favor of the intervention group compared to CON (Fig. 2A). We performed sensitivity analyses taking off studies that involved only RT (Fig. 2B) or RE (Fig. 2C) to clarify if the positive effects were due to RT or RE. Althought, both analyses showed significative effect in favor of the interventions, with mean difference of 3.97 (95% CI, 0.53 to 7.41, I^2^ = 0%; *p* = 0.02), considering studies with RE and 2.5 (95% CI, 0.87 to 4.13, I^2^ = 0%; *p* = 0.003), considering RT.

### Body composition

For the assessment of body composition, only one study used bioelectrical impedance analysis (BIA) to evaluate fat-free mass [29]. The intervention group (IG) showed a slightly improvement over the 24-week intervention, while the control group showed a decrease in fat-free mass. However, this difference between groups was not significant (Δ0.3 kg vs Δ-0.1 kg, *p* = 0.593). On the other hand, the authors demonstrated that the IG experienced a significant decrease in total body mass (*p* = 0.0001) and fat mass (*p* = 0.0149) compared to the control group.

### Physical performance

**Fig. 3:**
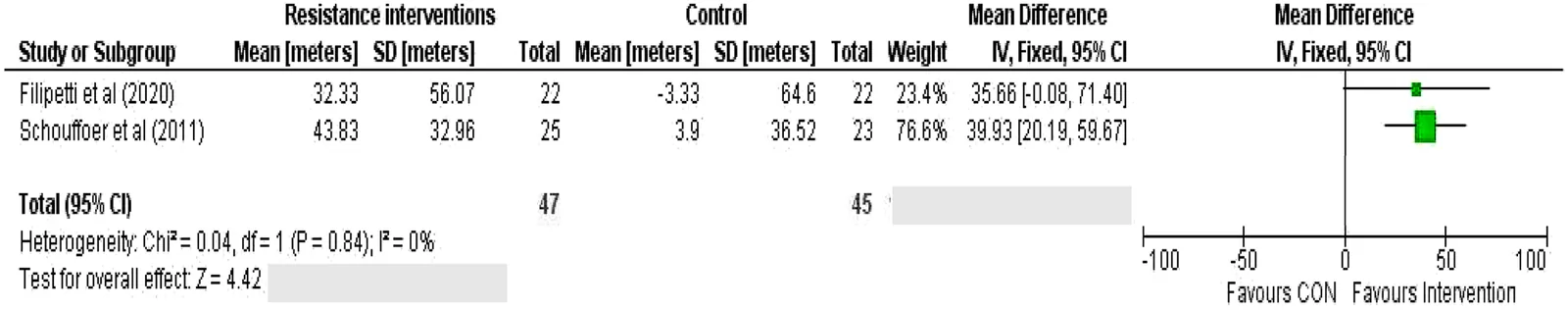
Effects of interventions on physical performance assessed by the 6-minute walk test. Abbreviations: CON: control; I2: inconsistency of studies; SD: standard deviation; MD: mean difference; 95% CI: 95% confidence interval; IV: inverse variance; fixed: fixed effects model

The physical performance was evaluated by the 6-minute walk test (6-MWT) in four of the included studies [29, 30, 37, 38]. One study assessed physical performance by the Canadian Occupational Performance Measurement (COPM) [35]. Of those who used the 6-MWT to assess physical performance, one [38] did not provide data from this assessment. We contacted the corresponding author, but he was unable to provide the data due to logistical difficulties. Two of the included studies had data extracted for meta-analysis comparing RE and CON on physical performance in SSc patients. It was not possible to present a quantitative comparison due to the small number of studies that evaluated physical performance. Although, we show a descriptive presentation of the single effects (Fig. 3). The pooled effect size, as well as the significance, were both omitted to avoid misleading interpretation of the results.

### Physical disability

**Fig. 4:**
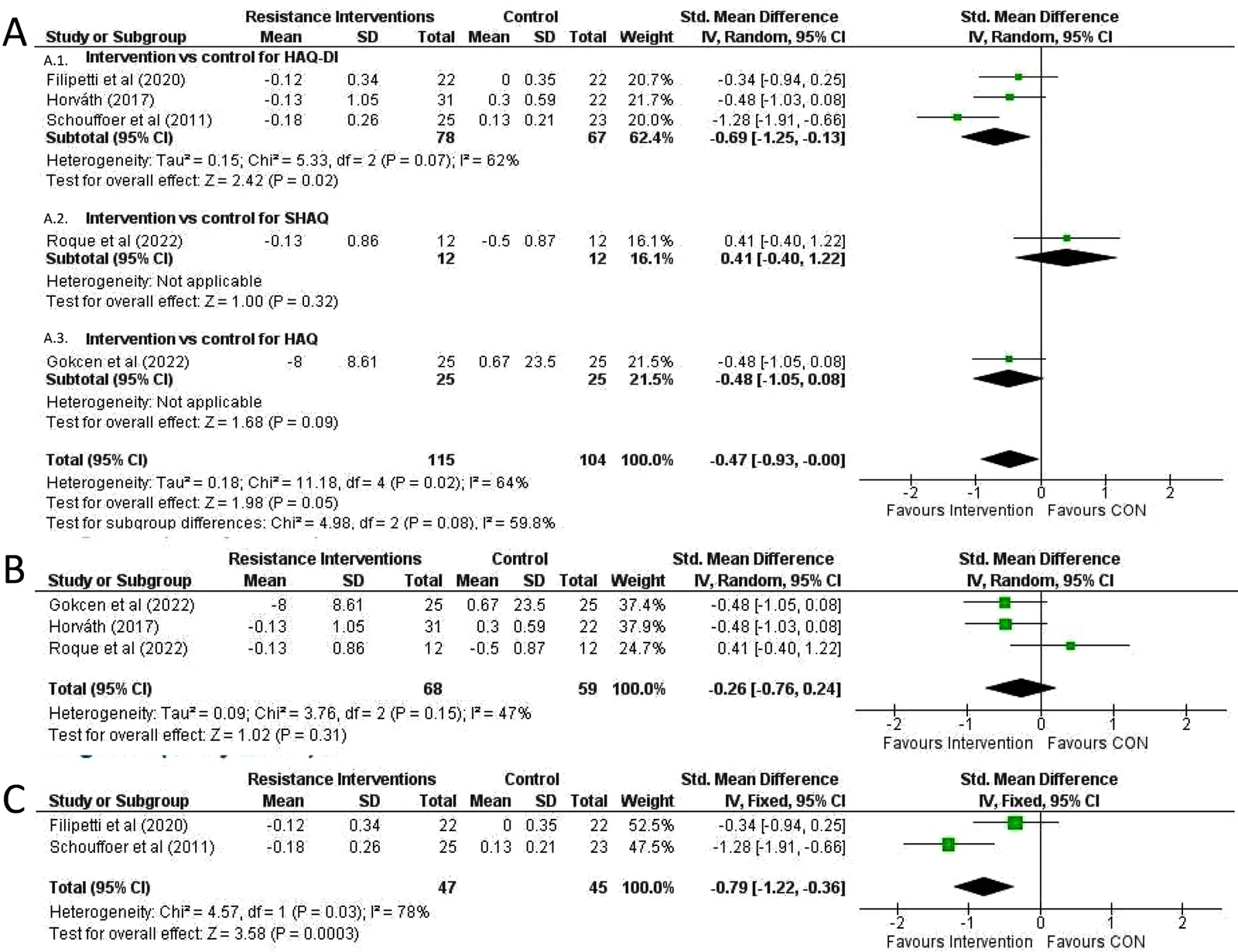
Effects of interventions on functional disability assessed by health assessment questionnaire and its different versions. **A**. Analysis with all studies that evaluated functional disability by HAQ. A.1. studies that evaluated functional disability by HAQ-DI. A.2. studies that evaluated functional disability by SHAQ. A.3. studies that evaluated functional disability by HAQ. **B**. Sensitivity analysis taking off studies that involved resistance training. **C**. Sensitivity analysis taking off studies that involved resistance exercises. Abbreviations: HAQ: health assessment questionnaire; SHAQ: HAQ for adapted for systemic sclerosis; HAQ-DI: HAQ disability index; CON: control; I2: inconsistency of studies; SD: standard deviation; SMD: standardized mean difference; 95% CI: 95% confidence interval; IV: inverse variance; Random: random effects model; fixed: fixed effects model

Most of included studies assessed physical disability by the health assessment questionnaire disability index (HAQ-DI) [29, 30, 32, 33, 37], two authors evaluated with the HAQ modified for SSc (SHAQ) [30, 34], one chose for evaluation with the HAQ [31], and one with the Disabilities of the Arm, Shoulder, and Hand Score (DASH) [30]. From the ten included studies, five had data extracted for meta-analysis comparing RE and CON on physical disability in SSc patients. We presented the meta-analysis (Fig. 4) with the studies that used the HAQ-DI (Fig. 4A), one study that performed the HAQ adapted for SSc (SHAQ) (Fig. 4B), and one study that performed the HAQ (Fig. 4C). Considering the five studies, the standardized mean difference was −0.47 in favor of the intervention group (95% CI, −0.93 to −0.00, I^2^ = 64%; *p* = 0.05). We performed sensitivity analyses taking off studies that involved only RT (Fig. 4B) or RE (Fig. 4C) to clarify if the positive effects were due to RT or RE. The difference was only significant, in favor of interventions, when we took off the RE, with a standardized mean difference of −0.79 (95% CI, −1.22 to −0.36, I^2^ = 64%; *p* = 0.05).

### Quality of life

**Fig. 5:**
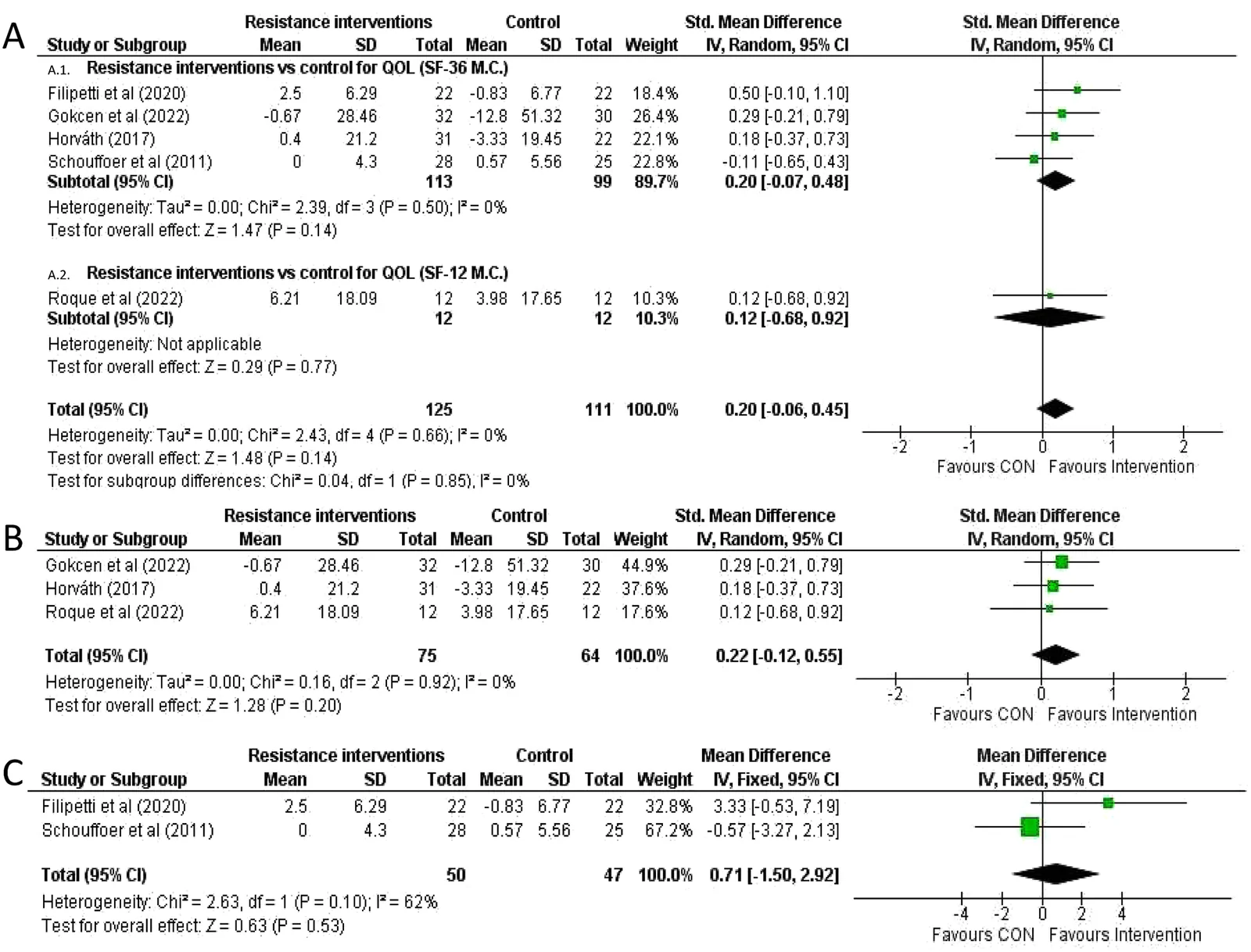
Effects of interventions on quality of life (mental component). **A**. Analysis with all studies that evaluated QOL. A.1. studies that evaluated QOL by SF-36. A.2. studies that evaluated QOL by SF-12. **B**. Sensitivity analysis taking off studies that involved resistance training. **C**. Sensitivity analysis taking off studies that involved resistance exercises. Abbreviations: HAQ: health assessment questionnaire; SHAQ: HAQ for adapted for systemic sclerosis; HAQ-DI: HAQ disability index; CON: control; I2: inconsistency of studies; SD: standard deviation; SMD: standardized mean difference; 95% CI: 95% confidence interval; IV: inverse variance; Random: random effects model; fixed: fixed effects model

**Fig. 6:**
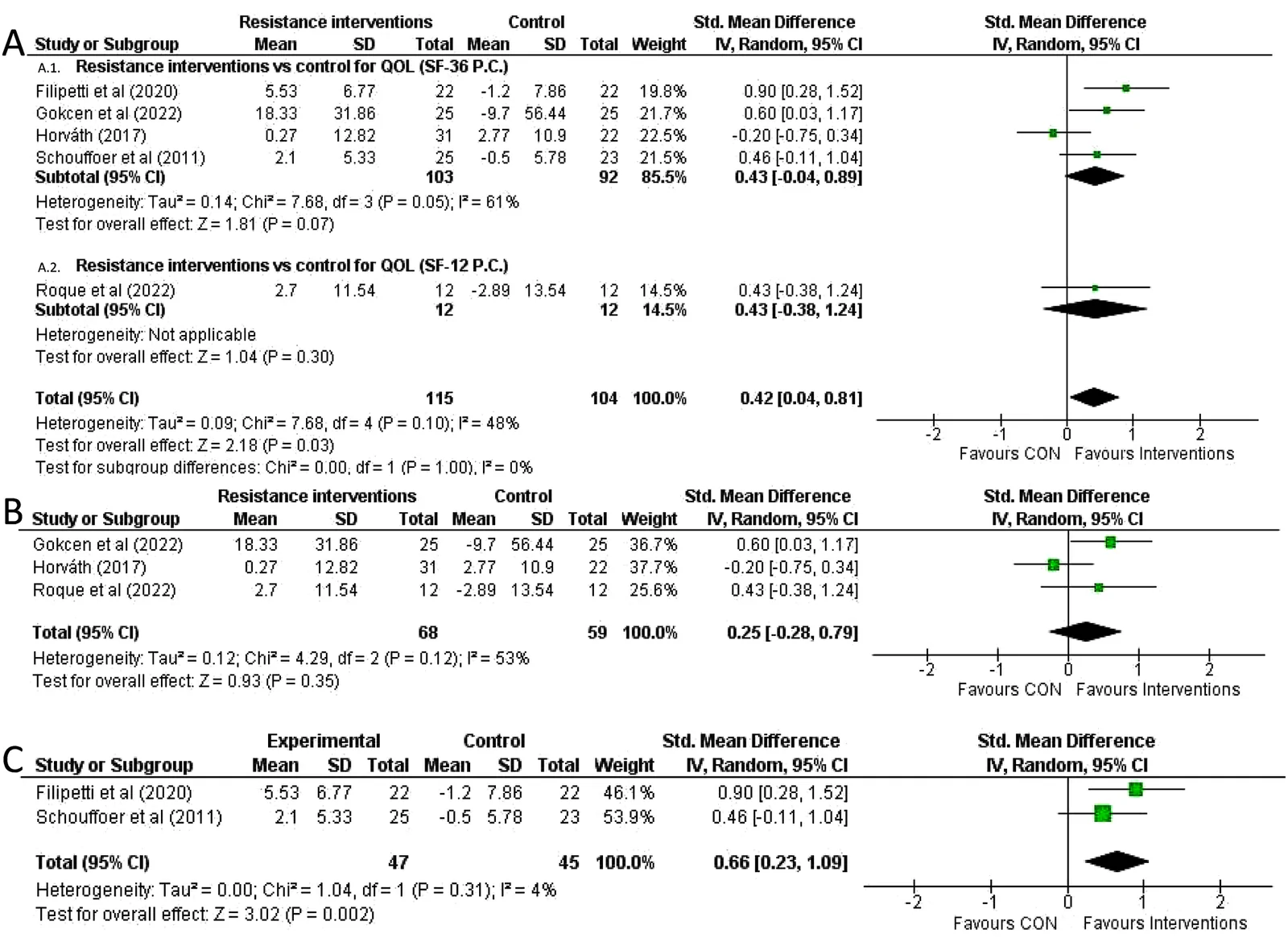
Effects of interventions on quality of life (physical component). **A**. Analysis with all studies that evaluated QOL. A.1. studies that evaluated QOL by SF-36. A.2. studies that evaluated QOL by SF-12. **B**. Sensitivity analysis taking off studies that involved resistance training. **C**. Sensitivity analysis taking off studies that involved resistance exercises. Abbreviations: HAQ: health assessment questionnaire; SHAQ: HAQ for adapted for systemic sclerosis; HAQ-DI: HAQ disability index; CON: control; I2: inconsistency of studies; SD: standard deviation; SMD: standardized mean difference; 95% CI: 95% confidence interval; IV: inverse variance; Random: random effects model; fixed: fixed effects model

In order to evaluate the influence of RE on quality of life, five studies provided data for a meta-analysis comparing to a CON. In these studies, four assessed the mental and physical health components of quality of life using the SF-36 [29, 31, 32, 37], one study used the SF-12 [34]. One study used the Nottingham Health Profile [36] and another study used the EQ-5D-5 L to assess the quality of life. We considered, for Table 2, the “usual activities” and “anxiety” domains of the EQ-5D-5 L questionnaire. We considered only those with similar scale (SF-36 and SF-12) for meta-analyses. The results of the meta-analyses are presented in Figs. 5 and 6

With regard to the mental component of quality of life, no significant differences were observed between the intervention group and CON (SMD = 0.20, 95% CI, −0.06 to 0.45, I^2^ = 0%; *p* = 0.14) (Fig. 5A). We performed sensitivity analyses taking off studies that involved only RT (Fig. 5B) or RE (Fig. 5C) to verify if either RT or RE would show significant effects, but the differences remained not significant. Moreover, the physical component of quality of life exhibited a positive outcome when interventions was contrasted with the CON (Fig. 6A: SMD = 0.42, 95% CI, 0.04 to 0.81, I^2^ = 48%; *p* = 0.03). In a sensitivity analysis designed to verify the effects of RE or RT, we took off RT (Fig. 6B), and the difference between RE and CON was not significant. Conversely, when we took off RE, the analysis presented significant difference in favor of the intervention group, with a standardized mean difference of 0.66 (95% CI, 0.23 to 1.09 I^2^ = 4%; *p* = 0.002).

### Adherence and safety

Adherence details were reported for all studies, with the majority exceeding the 10% of dropouts estimated in the sample calculation. Sari et al. (2023) [36] reported a dropout rate of 9,2%, with 46 out of 50 patients completing the study. Yakut et al. (2021) [30] reported dropout rates of about 10%, with 37 out of 40 patients completing the intervention. The lowest rate of adherence was reported by Filippetti et al. (2020) [29], 33 out of 44 patients completed the study, indicating a 25% dropout rate. Gokcen et al. (2022) [31] reported that 12 out of 62 patients were lost to follow-up, indicating a 19.3% dropout rate. Roque et al. (2022) [34] reported that 4 out of 24 patients did not complete the weeks of training, indicating a 16.6% dropout rate. Piga et al. (2014) [33] reported a mean dropout rate of 17,5% across groups. Sahin et al. (2023) [35] reported that 9 out of 55 patients were lost to follow-up, indicating a 16.3% dropout rate. Schouffoer et al. (2011) [37] reported that 6 out of 53 patients were lost to follow-up, indicating a 11.3% dropout rate. Only three studies reported a rate of exercise compliance. Sahin et al. (2023) [35] observed 87.1% in the rate of exercise compliance. Gokcen et al. (2022) [31] reported a median of 81%, and Schouffoer et al. (2011) [37] reported more than 80% in both groups.

From the ten included studies, 6 [29–31, 33, 35, 36] studies did not report adverse effects. Furthermore, none of the studies demonstrated a correlation between adverse effects and the course of the disease. Schouffoer et al. (2011) [37] reported that two patients withdrew due to adverse effects after 3 weeks of participation; however, another patient experienced an Achilles tendon rupture during circuit training in the second week. During bicycle training, 6 patients in the intervention group trained with an intensity lower than 60% of the specific maximum heart rate due to pain. Roque et al. (2022) [34] reported pain in seven patients and hand fatigue after exercise in five participants. These symptoms were mentioned in the initial weeks, ceasing to be reported approximately between the sixth and seventh week of treatment.

### Methodological quality assessment

Most included studies ranged from good [30, 31, 36, 37] to excellent [29, 34, 38], and three of them [32, 33, 35] were considered as regular in accordance with the PEDro scale. The complete pairwise analysis is available at supplemental digital content 4

### Risk of bias

**Fig. 7:**
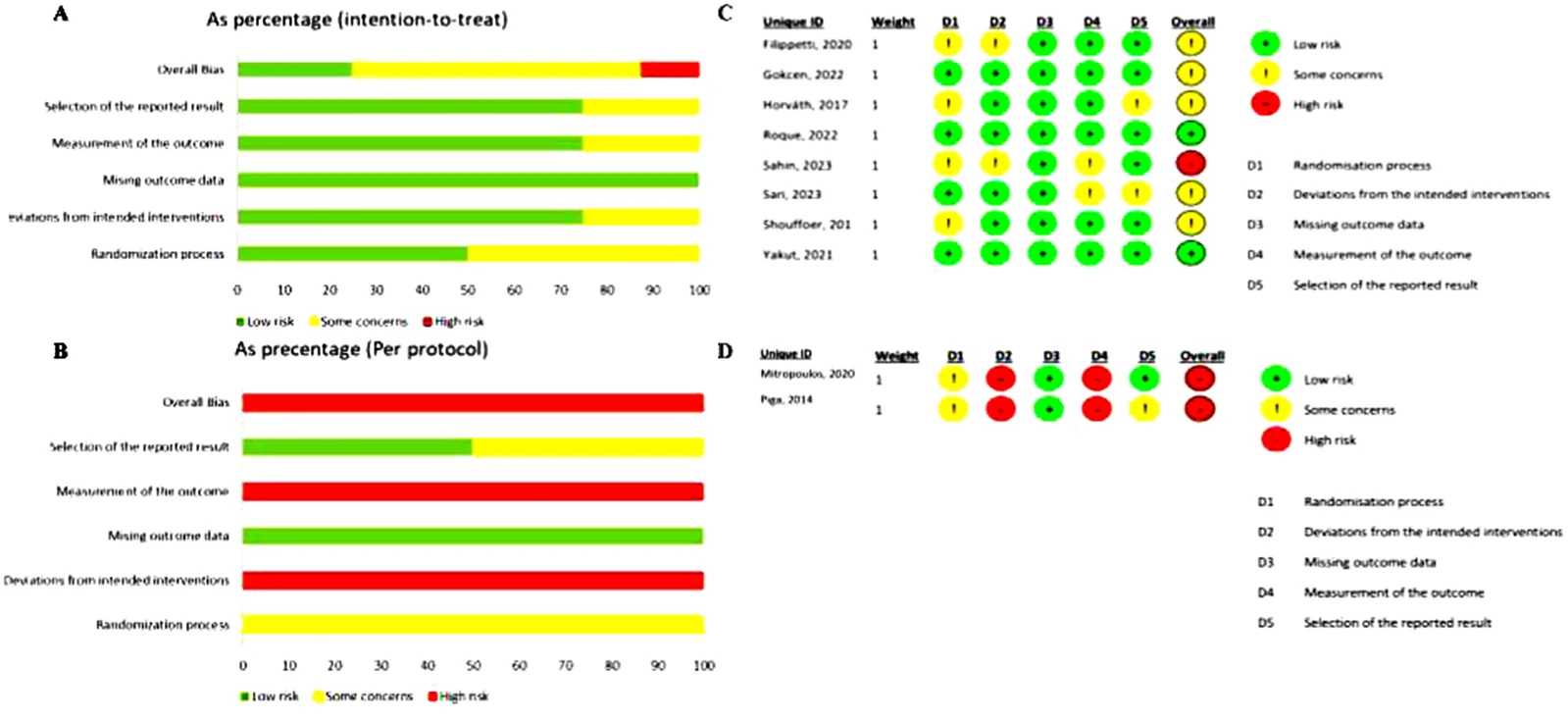
Risk of bias graph considering all studies pooled. **A**: overall risk of bias assessment for all included papers at the intention-to-treat (ITT) assessment; **B**: overall risk of bias assessment for all included studies at per protocol (PP) assessment; **C**: risk of bias for each of the five domains for the included papers at the ITT; **D**: risk of bias for each of the five domains for the included papers at the PP; D1 to D5: domains 1 to 5

From ten included studies, 5 [29, 31, 40] presented some concerns and three [33, 35, 38] presented high risk for the overall bias. The complete assessment is available on Fig. 7 Fig. 7(A) presents the overall risk of bias assessment for all included studies at intention-to-treat (ITT) and 7(B) at the per-protocol (PP) assessment methods. Figure 7(C) presents the risk of bias for each of the five domains for the included studies at the ITT and 7(D) at the PP method. The complete peer review evaluation is presented at supplemental digital content 5

### Quality of evidence and methodological quality

Considering the quality of evidence, the GRADE assessment showed a low quality of evidence for muscle strength assessment and very low quality of evidence for physical disability and QOL (both mental and physical components). The complete peer review assessment is presented at supplemental digital content 6

## Discussion

Our main findings from this systematic review with meta-analysis indicate that interventions with RE improved muscle strength, physical disability, and the physical component of QOL in SSc patients compared to the control group. When performing sensitivity analysis, RT showed better efficacy than RE to improve functional disability and QOL. There were no differences in the mental component of QOL. Regarding body composition and physical performance, since the number of studies that could be included in a meta-analysis was limited. Although a previous systematic review [18] had a similar objective to ours by summarizing the effects of physical therapy in patients with SSc, this is the first systematic review specifically aimed at summarizing the effects of interventions with RE compared to other exercise modalities and controls on specific outcomes, including adherence, in patients with SSc.

Although it is established that interventions with RE enhance strength in diverse populations, there is a paucity of data on SSc. Given the gravity of this disease, which encompasses involvement of the locomotor system, it is imperative to ascertain the effects of this intervention on muscle strength. Previous literature already shows that patients diagnosed with SSc, frequently exhibit diminished muscle strength, limited physical activity and low exercise engagement [3, 18]. This reduction in muscle strength appears to significantly impact their physical disability and QOL in SSc patients [41]. Additionally, it is important to observe that previous literature already demonstrated that handgrip strength is associated with lower risk of mortality in chronic diseases [41], which may be a reason to recommend RE for SSc patients. In our systematic review, considering included studies, it was possible to note that only four of them [29, 30, 37, 38] controlled training structure with objective measures, which we considered as RT. However, only two of those studies were included in meta-analyses, since the study from Yakut et al. (2021) [30], compared two different RT protocols (supervised vs home-based) and showed significant difference between groups for handgrip strength after intervention period (*p* < 0.001). On the other hand, the study from Piga et al. (2014) [33], which compared two interventions without supervision and with similar exercise protocols, did not show significant difference between groups for any of the muscle strength assessments. In addition, when performing sensitivity analyses, both RE and RT showed significant differences compared to the control group, suggesting that interventions with RE may be a useful non-pharmacological strategy to improve strength in SSc patients whether or not they had a well-structured training program. These findings are in line with previous literature that demonstrated signigicant improvement of muscle strength in rheumatic diseases patients who underwent RT [42] and RE [43], considering our definition of these two kinds of intervention.

Regarding the meta-analysis evaluating physical disability, patients who underwent interventions with RE exhibited significant improvements compared to those in the CON group. We performed subgroup analyses to elucidate the effects of RE on physical disability, separating the studies based on the use of different questionnaires: HAQ-DI [29, 32, 37], SHAQ [34], and HAQ total [31]. Interestingly, when separated by the HAQ version, both SHAQ and HAQ total did not show significant differences between groups. To clarify these findings, we performed sensitivity analysis excluding either RT or RE from the metanalysis and only when considering studies with rigorous, structured and progressive RT protocol [29, 30, 37, 38], the difference between groups was significant, suggesting that the periodization of training program may be the key point to improve functional disability. Additionally, the study from Yakut et al. (2021) [30] have demonstrated that RT leads to a significant improvement in HAQ compared to other protocols, with higher intensities being necessary for greater efficacy. Furthermore, a previous systematic review emphasized the use of non-pharmacological therapies to improve hand function and activities of daily living in patients with SSc [12] RE Our findings demonstrated that interventions with RE may be effective to improve QOL, considering the physical domain. We performed sensitivity analyses, considering only RE or RT to verify if any of those would be better to improve the physical domain of QOL, and the studies that performed a structured RT [29, 30, 37, 38] presented significant differences between groups for this outcome. On the other hand, studies that performed RE [31–36] did not show significant differences between groups. Two studies [36, 38] assessed QOL through different tools - the NHP and the EQ-5D-5 L - and the intervention groups from both studies showed significant differences for the QOL improvement compared to the CON. Despite the fact that we did not consider the study from Sari et al., 2023 [36] for the RT group, it is important to note that the intervention group of this study underwent “clinical Pilates”, which may be considered as RT but, as the protocol was not clear, we considered it as RE. These findings corroborate a previous systematic review [44] that analyzed the effects of resistance training (RT) on general health-related QOL in patients with rheumatic diseases, which included 32 studies in qualitative synthesis and 29 for the quantitative synthesis and found significant difference in favor of RT when compared to control group. Additionally, when compared to other exercise interventions, there were no significant differences, even with low intensity RT, which demonstrates that, even with low intensity, interventions with RE could improve the general QOL in patients with rheumatic diseases. Lastly, they did not find significant differences between groups for the individual components of SF-36 (mental and physical components), which is partly in line with our findings, since we found significant differences between groups for the physical component of QOL.

Only one study [29] assessed body composition by fat-free mass evaluation and did not show significant difference between RE vs CON groups. Regarding physical performance, although a statistical comparison between RE and CON was not feasible, the results suggest that RE has a clinically meaningful impact on physical performance. Mitropoulos et al. (2020) stated in the methods section that they would assess physical performance using the 6MWT. However, these data were not provided in the paper or elsewhere. We contacted the corresponding author to obtain these data, but he was unable to provide them. Our findings are consistent with the findings of a previous systematic review [44], which demonstrated a significant improvement in the 6 Minute Walk Test (6MWT) with RE in patients with chronic obstructive pulmonary disease. Thus, we speculate that RE may be a valuable addition for health professionals prescribing therapy or training for SSc patients.

Seven studies in this review provided adherence data [29–31, 33, 34, 36, 38]. Among these studies, only three studies [29, 30, 38] reported adherence consistent with the expected sample size, while most studies in this review had higher dropout rates. This underscores the challenge of maintaining patients in the prescribed exercise program, a difficulty also noted in a prior systematic review with SSc patients [45]. Studies with SSc patients should prioritize adherence monitoring, considering that assessing patient adherence is vital for improving exercise prescriptions, especially for populations like the elderly [46]. Additionally, the negative impacts of the RE regimens remain unclear due to the lack of standardized evaluation methods. Ensuring good adherence to these exercises is crucial for improving the quality of life (QOL), cardiovascular function, metabolic and glandular health, muscle structure and function, lung mechanics, mobility, and reducing systemic inflammation in patients with systemic sclerosis (SSc). These exercises have beneficial effects on both the physiological and psychological components of the condition [46].

Despite finding a significant effect of interventions with RE on muscle strength, physical disability, and physical component of QOL, caution is warranted when applying these interventions to SSc patients, due to the moderate to high risk of bias assessed in most included studies and the very low quality of evidence for the main outcomes. The absence of standardization presents a significant challenge when attempting to perform statistical analysis across multiple studies. The integration of both quantitative and qualitative analyses can become complex when studies employ disparate evaluation methodologies. The heterogeneity of the intervention protocols in the included studies presents challenges for health professionals in offering interventions with RE as a strategy for SSc patients. Additionally, the reproducibility of the RCTs is hindered by the lack of clarity surrounding the exercise variables in most of the studies. The findings of this study suggest the potential efficacy of interventions incorporating RE in enhancing muscle strength, irrespective of the presence of a structured exercise program. On the other hand, when considering functional disability and quality of life, our results suggest that only structured RT was able to improve these outcomes. However, it is important to note that there were no trials with RT or RE that were not combined with other interventions, which may be a confounding factor, especially when considering functional disability and QOL, which may be influenced by interventions such as aerobic training, breathing and/or stretching exercises. Yet, differences between RT and RE may be related to physiological mechanisms. As mentioned above, muscle strength is dependent on exercise intensity, with recruitment of type IIa and IIx muscle fibres and larger motor units activated by a resistance stimulus [47]. In addition, these effects appear to be greater in the initial phase of the intervention [47] which is consistent with our findings in studies within 12 weeks of duration. Conversely, interventions with RE were only able to improve functional disability and QOL when the exercise protocol was well structured. These findings are in line with previous literature showing that structured resistance exercises combined with aerobic training can improve symptoms, functional disability and QOL in many populations [48–50]. Nevertheless, the physiological pathways remain not well described, but we speculate that it may be related to the systemic effects of a structured protocol, which may not be present in a protocol that only involves fingers, hands, fists and mouth exercises, as we may see in three included studies [31, 33, 34]. Previous literature has demonstrated that a structured RT produces a systemic metabolic stress, that may be useful for maintaining or even regaining health, with the anti-inflammatory effects (IL-6 and irisin), binding of myostatin by the follistatin and decorin and the production of BDNF by a variety of tissues, such as muscle, liver, adipose tissue and brain [47]. It has been suggested in the literature that, depending on the therapeutic target, the mTOR or AMPK pathways can be activated for myokine production [47]. Although there is a discussion about the role of myokines in rheumatic diseases, previous study suggests that muscle production of these cytokines may have an anti-inflammatory effect [51] which we understand could be an explanation for our findings, but as we said, it is not yet determined. Further research is required utilizing well-described interventions and assessment methods, with a comparison of two or more non-pharmacological interventions, and with one or more groups performing exclusively RT. This will facilitate a more comprehensive understanding of the effects of interventions with RE on muscle strength, body composition, physical performance, physical disability, and QOL in SSc patients. In addition, our findings support the EULAR recommendations for the use of RE as a treatment for SSc [40]. It is important to note that we standardized the duration of the interventions across the included studies to 12 weeks for the meta-analyses to ensure consistency in the statistical analysis.

The research has some limitations. Few RCTs compared interventions with RE to other non-pharmacological modalities, as seen in other populations (e.g. older individuals or RA) [52, 53]. Most of the studies included [31, 34, 35, 37] did not utilize machines or free weights, which are commonly used and studied in other populations, such as older individuals and those with other rheumatic conditions [54, 55] In the majority of studies, the interventions with RE were combined with other exercise interventions. This methodological choice limits the possibility of analyzing the REs effect alone or describing the physiological explanation for its effects on outcomes. However, we hypothesized that RT may be a better strategy to improve muscle strength, functional disability and QOL in SSc patients, according to our findings. Finally, it was not possible to perform meta-analyses considering the body composition and physical performance in SSc patients, due to the low number of studies that assessed these outcomes.

Our findings suggest that interventions with RE may be a safe and effective non-pharmacological strategy, demonstrating significant improvements in physical capacities such as muscle strength, physical function, and the physical component of quality of life in patients with SSc. Given the impairing nature of SSc and the concomitant concerns regarding its severity and the practice of high-intensity resistance training [56, 57], it was deemed worthwhile to broaden our evaluation to encompass interventions with RE, as well as its safety. Furthermore, our review suggests that RT may be the most effective non-pharmacological intervention, demonstrating the importance of controlling training variables such as intensity, volume, density, frequency and duration of exercise programmes. We believe that SSc patients would benefit of a well-structured RT, considering our findings and previous literature. These results provide health professionals with multiple intervention options, allowing them to consider factors such as aerobic limitations, nutritional and inflammatory status, functional capacity, and disease severity, rather than adopting a one-size-fits-all approach for patients with SSc.

Our results suggest that interventions with RE may be a useful addition to the treatment of patients with systemic sclerosis, in improving muscle strength, physical disability and the physical component of quality of life. The best intervention with RE protocol for patients with SSc remains undefined. Different protocols should prioritize individual aspects such as aerobic limitations, nutritional and inflammatory status, functional capacity, and disease severity, rather than adopting a one-size-fits-all approach for patients with systemic sclerosis.

## Practical applications

The results of this study suggest that incorporating structured resistance training into rehabilitation programmes for patients with SSc can effectively improve muscle strength, reduce physical disability and improve the physical component of QoL. Healthcare professionals should consider personalised exercise prescriptions that take into account individual factors such as aerobic capacity, inflammation levels and disease severity to optimise outcomes. In addition, the results support the development of specific exercise guidelines for SSc and highlight the importance of monitoring adherence to maintain the benefits of RT, in line with EULAR recommendations for non-pharmacological interventions.

## Electronic supplementary material

Below is the link to the electronic supplementary material.

Supplementary Material 1

Supplementary Material 2

Supplementary Material 3

Supplementary Material 4

Supplementary Material 5

Supplementary Material 6

## Data Availability

The datasets used and/or analysed during the current study are available from the corresponding author on reasonable request.
