# Supplementary material for: Effects of interventions with resistance exercises on muscle strength, physical disability, and quality of life in systemic sclerosis patients: a systematic review with meta-analysis
Source: Adv Rheumatol. 2025 Jul 28;65:34. doi: 10.1186/s42358-025-00468-1 (PMC13488637; doi:10.1186/s42358-025-00468-1)
Supplement: Supplementary file 2 [file 42358_2025_468_MOESM2_ESM.pdf]

#	PICO		
#1	P	(Scleroderma, Systemic[mh] OR systemic sclero*[tiab] OR diffuse sclero*[tiab] OR limited sclero*[tiab] OR CREST Syndrome[tiab] OR CRST Syndrome[tiab] OR Progressive Sclero*[tiab] OR Sudden Onset Sclero*[tiab])	
#2	I	(Resistance Training[mh] OR Resistance Training*[tiab] OR Resistance exercise*[tiab] OR Strength Training*[tiab] OR Resistance Strength Training*[tiab] OR Resistance Strength Exercise*[tiab] OR Strength exercise*[tiab] OR Strengthening Program*[tiab] OR Strength Program*[tiab] OR Weight-Lifting[tiab] OR Weight-Bearing[tiab] OR Muscle Contraction[mh] OR Muscle Contraction*[tiab] OR Muscular Contraction*[tiab] OR Inotropi*[tiab] OR Isometric Contraction*[tiab] OR Isotonic Contraction*[tiab] OR Asthenia[mh] OR Astheni*[tiab] OR Rehabilitation[mh:noexp] OR Rehabilitation[tiab] OR Occupational Therapy[mh] OR Occupational Therap*[tiab] OR Ergotherap*[tiab] OR Exercise Therapy[mh] OR Exercise Therap*[tiab] OR Remedial Exercise*[tiab])	
#3	#1 AND #2		188
