# Supplementary material for: Effects of interventions with resistance exercises on muscle strength, physical disability, and quality of life in systemic sclerosis patients: a systematic review with meta-analysis
Source: Adv Rheumatol. 2025 Jul 28;65:34. doi: 10.1186/s42358-025-00468-1 (PMC13488637; doi:10.1186/s42358-025-00468-1)
Supplement: Supplementary file 3 [file 42358_2025_468_MOESM3_ESM.pdf]

| Authors (year) | Group | Descrição dos treinamentos | Duration (weeks) | Times (weeks) | Frequency (sessions per week) | Sets per exercise | Reps per set | Interval (seconds) | Intensity (%1RM) | Intensity (RPE, 0-10) |
|---|---|---|---|---|---|---|---|---|---|---|
| Yakut et al (2021) | SEG | Combined exercise training (breathing + RT + AT) under supervision | 12 | 12 | 2 | 1 | 8-12 | 120 | 40-80 | 3-6 |
| | HEG | Home-based Breathing + posture + walking without supervision | 12 | 12 | 2 | 1 | 8-12 | | | 3-6 |
| Filippetti et al (2020) | IG | Home-based combined exercise training (RT + AT) without supervision | 24 | 12, 24 | - | 2 | 10 | 120 | 60 | - |
| | CG | Phone calls to evaluate the health status | 24 | 12, 24 | - | - | - | - | - | - |
| Schouffoer et al (2011) | IG | General exercises (AT + RT), hand/mouth exercises, and educational sessions | 12 | 12 | 1 | - | 8-15 | - | - | - |
| | CG | Usual outpatient care | - | 12 | - | - | - | - | - | - |
| Roque et al (2022) | IG | Free active and resisted active exercises (fingers, fist and hand grip strength) for both hands | 12 | 12 | 2 | - | - | - | - | - |
| | CG | Booklet with information about the disease | - | 12 | - | - | - | - | - | - |
| Gokcen et al (2022) | IG | 1-hand exercise training (isometric exercise with an exercise ball and self-administered stretching) with supervision | 8 | 8 | 7 | 3 | 15 | - | - | - |
| | CG | Education about SSc + booklet with recomedations | - | 8 | - | - | - | - | - | - |
| Sahin et al (2023) | IG | Upper extremity home exercises (stretching and strengthening) | 8 | 8 | 5 | 2 | 10 | - | - | - |
| | CG | Patient education | 8 | - | - | - | - | - | - | - |
| Horváth et al (2017) | IG | complex physiotherapy program including thermal baths medical massages of upper extremities exercise (30 minutes/day, isometric, isotonic and stretching hand exercises; ergotherapy | 3 | 3 | 5 | - | - | - | - | - |
| | CG | The control group received similar ther-apy to their large joints, and the spine without being a subject to any treat-ment to the hands, | 3 | 3 | 5 | - | - | - | - | - |

| | | | | | | | | | | |
|---|---|---|---|---|---|---|---|---|---|---|
| Mitropoulos et al (2019) | IG | The HIIT protocol, With respect to the RT, patients performed five upper body exercises in a circuit row for three circles interspersed | 24 | 24 | 2 | 3 | 10 | 30 | - | - |
| | CG | Control group did not perform the exercise programme, | 24 | 24 | 0 | 0 | 0 | 0 | - | - |
| Piga et al (2014) | IG | Hand kynesiotherapy exercises with a portable device, It consisted of 4 strengthening and 3 mobility exercises, | 12 | 12 | 5 | 1-2 | 5-20 | - | - | Painless maximum intensity |
| | CG | Hand kynesiotherapy exercises with common daily use objects, It consisted of 3 strengthening and 3 mobility exercises, | 12 | 12 | 5 | 1-2 | 5-20 | - | - | Painless maximum intensity |
| Sari (2023) | IG | Clinical Pilates-based exercises were given to the telerehabilitation-based treatment | 8 | 8 | 7 | 2 | 1 | - | | |
| | CG | Orientation: Electronic supplementary material. The exercises given to the patientsin a home program were the same asthose in the telerehabilitation-based exercise program. | 8 | 8 | 7 | 2 | 1 | - | | |
| Mitropoulos (2020) | IG | Combined exercise training (HIIT + RE) under supervision | 24 | 12,24 | 2 | 3 | 10 | 120 - 180 | 10RM | - |
| | CG | Control group did not perform the exercise programme, | 24 | 12,24 | 0 | 0 | 0 | 0 | 0 | - |
