# Supplementary material for: Effects of interventions with resistance exercises on muscle strength, physical disability, and quality of life in systemic sclerosis patients: a systematic review with meta-analysis
Source: Adv Rheumatol. 2025 Jul 28;65:34. doi: 10.1186/s42358-025-00468-1 (PMC13488637; doi:10.1186/s42358-025-00468-1)
Supplement: Supplementary file 4 [file 42358_2025_468_MOESM4_ESM.pdf]

PEDro peer review

Mayra

Author (Year)	Títulos	1	Onde	2	Onde	3	Onde	4	Onde	5	Onde	6	Onde	7	Onde	8	Onde	9	Onde	10	Onde	11	Onde	TOTAL
Filippetti (2020)	Effect of a tailored home-based exercise program in patients with systemic sclerosis: A randomized controlled trial	1	MATERIALS AND METHODS	1	MATERIALS AND METHODS	1	MATERIALS AND METHODS	1	MATERIALS AND METHODS	1	MATERIALS AND METHODS	1	MATERIALS AND METHODS	1	MATERIALS AND METHODS	1	Statistical analysis	1	Statistical analysis	1	Statistical analysis	1	Statistical analysis	10
Gokcen (2022)	The Efficacy of a Home-Based, Self-Administered Hand Exercise Program for Patients With Systemic Sclerosis	1	Study Design and Study Population	1	Randomization and Allocation Concealment	1	Randomization and Allocation Concealment	1	Study Design and Study Population	1	Randomization and Allocation Concealment	1	Randomization and Allocation Concealment	0	Intervention	0	Results	1	Intervention	1	Statistical Analysis	1	Sample Size	8
Roque (2022)	Joint mobilization and therapeutic exercises in the hands of patients with systemic sclerosis: A preliminary randomized controlled trial	1	Methods	1	Methods	1	Methods	1	Methods	1	Methods	0	Methods	1	Methods	1	Results	1	Results	1	Methods	1	Results	9
Schouffoer	Randomized Comparison of a	1	Patients.	1	Study design.	0	Patients.	1	Patients.	0	Study design.	0	PATIENTS AND	1	Assessments.	1	Patient inclusion	0	Patient inclusio	1	Statistical	1	Statistical	6

(2011)	Multidisciplinary Team Care Program With Usual Care in Patients With Systemic Sclerosis												METHODS				and characteristics.		n and characteristics.		analysis.		analysis.	
Yakut (2021)	Effects of supervised exercise program and home exercise program in patients with systemic sclerosis: A randomized controlled trial	1	Inclusion criteria Exclusion criteria	1	Study design	1	Study design	1	Measurements	0	Study design	0	Study design	0	Study design	1	Participants	0	Participants	1	Statistical analysis	1	Statistical analysis	6
Sahin (2023)	Investigation of the effects of upper extremity home exercises on grip strength, range of motion, activity performance, and functionality in individuals with systemic sclerosis: a randomized controlled trial	1	Participants	1	Participants	0	Participants	1	RESULTS	0	Participants	0	Outcome measures	0	Outcome measures	0	RESULTS	1	RESULTS	1	Statistical analysis	1	Statistical analysis	5
Horvat, 2017	Efficacy of intensive hand physical therapy in patients with systemic sclerosis	1	Recruitment and participants	0	Patients and methods Study	0	Patients and methods	1	Recruitment and participants	0	Recruitment and participants	0	Recruitment and participants	1	Outcomes of the study	1	Results	0	Results	1	Statistical analysis	1	Results	5

					design																		
Piga,2014	Telemedicine Applied to Kinesiotherapy for Hand Dysfunction in Patients with Systemic Sclerosis and Rheumatoid Arthritis: Recovery of Movement and Telemonitoring Technology	1	Preliminary evaluation, eligibility, and consent.	1	The experimental arm	0	The experimental arm	1	The experimental arm	0	The experimental arm	0	The experimental arm	0	RESULTS	1	RESULTS	0	RESULTS	1	Statistical analysis.	1	RESULTS	5
Sari, 2023	Effects of a telerehabilitation-based exercise program in patients with systemic sclerosis	1	Study design and ethics	1	Study design and ethics	0	Study design and ethics	1	Study design and ethics	0	Study design and ethics	0	Study design and ethics	0	Study design and ethics	1	Study design and ethics	1	Results	1	Statistical analysis	1	Results	6

Laura

Author (Year)	Títulos	1	Onde	2	Onde	3	Onde	4	Onde	5	Onde	6	Onde	7	Onde	8	Onde	9	Onde	10	Onde	11	Onde	TOTAL
Filippetti (2020)	Effect of a tailored home-based exercise program in patients with systemic sclerosis: A	1	MATERIALS AND METHODS	1	MATERIALS AND METHODS	1	MATERIALS AND METHODS	1	MATERIALS AND METHODS	1	MATERIALS AND METHODS	1	MATERIALS AND METHODS	1	MATERIALS AND METHODS	1	Statistical analysis	1	Statistical analysis	1	DISCUSSION	1	DISCUSSION	10

	randomized controlled trial																							
Gokcen (2022)	The Efficacy of a Home-Based, Self-Administered Hand Exercise Program for Patients With Systemic Sclerosis	1	Study Design and Study Population	1	Study Design and Study Population	1	Randomization and Allocation Concealment	1	Study Design and Study Population	1	Randomization and Allocation Concealment	1	Randomization and Allocation Concealment	0	Intervention	0	Sample Size	1	Intervention	1	RESULTS	1	RESULTS	8
Roque (2022)	Joint mobilization and therapeutic exercises in the hands of patients with systemic sclerosis: A preliminary randomized controlled trial	1	Methods	1	Methods	1	Methods	1	Results	1	Methods	0	Methods	1	Methods	1	Results	1	Methods	1	Discussion	1	Discussion	9
Schouffoer (2011)	Randomized Comparison of a Multidisciplinary Team Care Program With Usual Care in Patients With Systemic Sclerosis	1	Patients	1	Study design	0	Pacients	1	Pacients	0	Study Design	0	Multidisciplinary team care program (intervention)	1	Assessments.	0	Statistical analysis.	1	Study design	1	End points of effectiveness	1	End points of effectiveness	6

Yakut (2021)	Effects of supervised exercise program and home exercise program in patients with systemic sclerosis: A randomized controlled trial	1	Participants	1	Study design	1	Study design	1	Participants	0	Study Design	0	Study Designe Limitations and implications for future research	0	Study Design	1	Statistical analysis	0	Participants	1	RESULTS	1	RESULTS	6
Sahin (2023)	Investigation of the effects of upper extremity home exercises on grip strength, range of motion, activity performance, and functionality in individuals with systemic sclerosis: a randomized controlled trial	1	Participants	1	Participants	0	Participants	1	Results	0	Protocols	0	Protocols	0	-	0	Results	1	Results	1	RESULTS	1	RESULTS	5
Horváth (2017)	Efficacy of intensive hand physical therapy in patients with systemic sclerosis	1	Patients and methods	0	Patients and methods	0	Patients and methods	1	Results	0	Patients and methods	0	*Patients and methods*	1	Patients and methods	1	Patients and methods	0	Results	1	RESULTS	1	RESULTS	5

Piga (2014)	Telemedicine Applied to Kinesiotherapy for Hand Dysfunction in Patients with Systemic Sclerosis and Rheumatoid Arthritis: Recovery of Movement and Telemonitoring Technology	1	MATERIALS AND METHODS	1	MATERIALS AND METHODS	0	MATERIALS AND METHODS	1	MATERIALS AND METHODS	0	MATERIALS AND METHODS	0	MATERIALS AND METHODS	0	MATERIALS AND METHODS	1	Results	0	Results	1	MATERIALS AND METHODS	1	Results	5
Sari (2023)	Effects of a telerehabilitation-based exercise program in patients with systemic sclerosis	1	Materials and methods	1	Materials and methods	0	Materials and methods	1	Materials and methods	0	Materials and methods	0	Materials and methods	0	Materials and methods	1	Materials and methods	1	Results	1	Materials and methods	1	Results	6
