# Supplementary material for: Effects of interventions with resistance exercises on muscle strength, physical disability, and quality of life in systemic sclerosis patients: a systematic review with meta-analysis
Source: Adv Rheumatol. 2025 Jul 28;65:34. doi: 10.1186/s42358-025-00468-1 (PMC13488637; doi:10.1186/s42358-025-00468-1)
Supplement: Supplementary file 5 [file 42358_2025_468_MOESM5_ESM.pdf]

**Unique ID**	filippetti2020	**Study ID**		**Assessor**	DANIEL
**Ref or Label**		**Aim**	assignment to intervention (the 'intention-to-treat' effect)		
**Experimental**		**Comparator**		**Source**	Journal article(s)
**Outcome**		**Results**		**Weight**	1

Domain	Signalling question	Response	Comments
**Bias arising from the randomization process**	1.1 Was the allocation sequence random?	Y	
	1.2 Was the allocation sequence concealed until participants were enrolled and assigned to interventions?	NI	
	1.3 Did baseline differences between intervention groups suggest a problem with the randomization process?	N	
	**Risk of bias judgement**	**Some concerns**	
**Bias due to deviations from intended interventions**	2.1.Were participants aware of their assigned intervention during the trial?	Y	
	2.2.Were carers and people delivering the interventions aware of participants' assigned intervention during the trial?	Y	
	2.3. If Y/PY/NI to 2.1 or 2.2: Were there deviations from the intended intervention that arose because of the experimental context?	NI	
	2.4 If Y/PY to 2.3: Were these deviations likely to have affected the outcome?	NA	
	2.5. If Y/PY/NI to 2.4: Were these deviations from intended intervention balanced between groups?	NA	
	2.6 Was an appropriate analysis used to estimate the effect of assignment to intervention?	Y	
	2.7 If N/PN/NI to 2.6: Was there potential for a substantial impact (on the result) of the failure to analyse participants in the group to which they were randomized?	NA	
	**Risk of bias judgement**	**Some concerns**	
**Bias due to missing outcome data**	3.1 Were data for this outcome available for all, or nearly all, participants randomized?	Y	
	3.2 If N/PN/NI to 3.1: Is there evidence that result was not biased by missing outcome data?	NA	
	3.3 If N/PN to 3.2: Could missingness in the outcome depend on its true value?	NA	
	3.4 If Y/PY/NI to 3.3: Is it likely that missingness in the outcome depended on its true value?	NA	
	**Risk of bias judgement**	**Low**	
	4.1 Was the method of measuring the outcome inappropriate?	N	

**Bias in measurement of the outcome**	4.2 Could measurement or ascertainment of the outcome have differed between intervention groups?	N	
	4.3 Were outcome assessors aware of the intervention received by study participants?	N	
	4.4 If Y/PY/NI to 4.3: Could assessment of the outcome have been influenced by knowledge of intervention received?	NA	
	4.5 If Y/PY/NI to 4.4: Is it likely that assessment of the outcome was influenced by knowledge of intervention received?	NA	
	**Risk of bias judgement**	**Low**	
**Bias in selection of the reported result**	5.1 Were the data that produced this result analysed in accordance with a pre-specified analysis plan that was finalized before unblinded outcome data were available for analysis?	Y	
	5.2 ... multiple eligible outcome measurements (e.g. scales, definitions, time points) within the outcome domain?	N	
	5.3 ... multiple eligible analyses of the data?	N	
	**Risk of bias judgement**	**Low**	
**Overall bias**	**Risk of bias judgement**	**Some concerns**	

**Unique ID**	gokcen2022	**Study ID**		**Assessor**	DANIEL
**Ref or Label**		**Aim**	assignment to intervention (the 'intention-to-treat' effect)		
**Experimental**		**Comparator**		**Source**	Journal article(s)
**Outcome**		**Results**		**Weight**	1

Domain	Signalling question	Response	Comments
**Bias arising from the randomization process**	1.1 Was the allocation sequence random?	Y	
	1.2 Was the allocation sequence concealed until participants were enrolled and assigned to interventions?	Y	
	1.3 Did baseline differences between intervention groups suggest a problem with the randomization process?	N	
	**Risk of bias judgement**	**Low**	
	2.1.Were participants aware of their assigned intervention during the trial?	Y	
	2.2.Were carers and people delivering the interventions aware of participants' assigned intervention during the trial?	Y	

**Bias due to deviations from intended interventions**	2.3. If Y/PY/NI to 2.1 or 2.2: Were there deviations from the intended intervention that arose because of the experimental context?	N	
	2.4 If Y/PY to 2.3: Were these deviations likely to have affected the outcome?	NA	
	2.5. If Y/PY/NI to 2.4: Were these deviations from intended intervention balanced between groups?	NA	
	2.6 Was an appropriate analysis used to estimate the effect of assignment to intervention?	Y	
	2.7 If N/PN/NI to 2.6: Was there potential for a substantial impact (on the result) of the failure to analyse participants in the group to which they were randomized?	NA	
	**Risk of bias judgement**	**Low**	
**Bias due to missing outcome data**	3.1 Were data for this outcome available for all, or nearly all, participants randomized?	Y	
	3.2 If N/PN/NI to 3.1: Is there evidence that result was not biased by missing outcome data?	NA	
	3.3 If N/PN to 3.2: Could missingness in the outcome depend on its true value?	NA	
	3.4 If Y/PY/NI to 3.3: Is it likely that missingness in the outcome depended on its true value?	NA	
	**Risk of bias judgement**	**Low**	
**Bias in measurement of the outcome**	4.1 Was the method of measuring the outcome inappropriate?	PN	
	4.2 Could measurement or ascertainment of the outcome have differed between intervention groups?	N	
	4.3 Were outcome assessors aware of the intervention received by study participants?	NI	
	4.4 If Y/PY/NI to 4.3: Could assessment of the outcome have been influenced by knowledge of intervention received?	N	
	4.5 If Y/PY/NI to 4.4: Is it likely that assessment of the outcome was influenced by knowledge of intervention received?	NA	
	**Risk of bias judgement**	**Low**	
**Bias in selection of the reported result**	5.1 Were the data that produced this result analysed in accordance with a pre-specified analysis plan that was finalized before unblinded outcome data were available for analysis?	Y	
	5.2 ... multiple eligible outcome measurements (e.g. scales, definitions, time points) within the outcome domain?	N	
	5.3 ... multiple eligible analyses of the data?	N	
	**Risk of bias judgement**	**Low**	
**Overall bias**	**Risk of bias judgement**	**Some concerns**	

**Unique ID**	horvath2017	**Study ID**		**Assessor**	DANIEL
**Ref or Label**		**Aim**	assignment to intervention (the 'intention-to-treat' effect)		
**Experimental**		**Comparator**		**Source**	Journal article(s)
**Outcome**		**Results**		**Weight**	1

Domain	Signalling question	Response	Comments
**Bias arising from the randomization process**	1.1 Was the allocation sequence random?	N	
	1.2 Was the allocation sequence concealed until participants were enrolled and assigned to interventions?	N	
	1.3 Did baseline differences between intervention groups suggest a problem with the randomization process?	Y	
	**Risk of bias judgement**	**High**	
**Bias due to deviations from intended interventions**	2.1.Were participants aware of their assigned intervention during the trial?	Y	
	2.2.Were carers and people delivering the interventions aware of participants' assigned intervention during the trial?	Y	
	2.3. If Y/PY/NI to 2.1 or 2.2: Were there deviations from the intended intervention that arose because of the experimental context?	NI	
	2.4 If Y/PY to 2.3: Were these deviations likely to have affected the outcome?	NA	
	2.5. If Y/PY/NI to 2.4: Were these deviations from intended intervention balanced between groups?	NA	
	2.6 Was an appropriate analysis used to estimate the effect of assignment to intervention?	PY	
	2.7 If N/PN/NI to 2.6: Was there potential for a substantial impact (on the result) of the failure to analyse participants in the group to which they were randomized?	NA	
	**Risk of bias judgement**	**Some concerns**	
**Bias due to missing outcome data**	3.1 Were data for this outcome available for all, or nearly all, participants randomized?	N	
	3.2 If N/PN/NI to 3.1: Is there evidence that result was not biased by missing outcome data?	PN	
	3.3 If N/PN to 3.2: Could missingness in the outcome depend on its true value?	N	
	3.4 If Y/PY/NI to 3.3: Is it likely that missingness in the outcome depended on its true value?	NA	
	**Risk of bias judgement**	**Low**	

**Bias in measurement of the outcome**	4.1 Was the method of measuring the outcome inappropriate?	N	
	4.2 Could measurement or ascertainment of the outcome have differed between intervention groups?	PN	
	4.3 Were outcome assessors aware of the intervention received by study participants?	NI	
	4.4 If Y/PY/NI to 4.3: Could assessment of the outcome have been influenced by knowledge of intervention received?	Y	
	4.5 If Y/PY/NI to 4.4: Is it likely that assessment of the outcome was influenced by knowledge of intervention received?	NI	
	**Risk of bias judgement**	**High**	
**Bias in selection of the reported result**	5.1 Were the data that produced this result analysed in accordance with a pre-specified analysis plan that was finalized before unblinded outcome data were available for analysis?	Y	
	5.2 ... multiple eligible outcome measurements (e.g. scales, definitions, time points) within the outcome domain?	N	
	5.3 ... multiple eligible analyses of the data?	N	
	**Risk of bias judgement**	**Low**	
**Overall bias**	**Risk of bias judgement**	**High**	

**Unique ID**	roque2022	**Study ID**		**Assessor**	DANIEL
**Ref or Label**		**Aim**	assignment to intervention (the 'intention-to-treat' effect)		
**Experimental**		**Comparator**		**Source**	Journal article(s)
**Outcome**		**Results**		**Weight**	1

Domain	Signalling question	Response	Comments
**Bias arising from the randomization process**	1.1 Was the allocation sequence random?	Y	
	1.2 Was the allocation sequence concealed until participants were enrolled and assigned to interventions?	Y	
	1.3 Did baseline differences between intervention groups suggest a problem with the randomization process?	N	
	**Risk of bias judgement**	**Low**	

**Bias due to deviations from intended interventions**	2.1.Were participants aware of their assigned intervention during the trial?	Y	
	2.2.Were carers and people delivering the interventions aware of participants' assigned intervention during the trial?	Y	
	2.3. If Y/PY/NI to 2.1 or 2.2: Were there deviations from the intended intervention that arose because of the experimental context?	N	
	2.4 If Y/PY to 2.3: Were these deviations likely to have affected the outcome?	NA	
	2.5. If Y/PY/NI to 2.4: Were these deviations from intended intervention balanced between groups?	NA	
	2.6 Was an appropriate analysis used to estimate the effect of assignment to intervention?	Y	
	2.7 If N/PN/NI to 2.6: Was there potential for a substantial impact (on the result) of the failure to analyse participants in the group to which they were randomized?	NA	
	**Risk of bias judgement**	**Low**	
**Bias due to missing outcome data**	3.1 Were data for this outcome available for all, or nearly all, participants randomized?	Y	
	3.2 If N/PN/NI to 3.1: Is there evidence that result was not biased by missing outcome data?	NA	
	3.3 If N/PN to 3.2: Could missingness in the outcome depend on its true value?	NA	
	3.4 If Y/PY/NI to 3.3: Is it likely that missingness in the outcome depended on its true value?	NA	
	**Risk of bias judgement**	**Low**	
**Bias in measurement of the outcome**	4.1 Was the method of measuring the outcome inappropriate?	N	
	4.2 Could measurement or ascertainment of the outcome have differed between intervention groups?	N	
	4.3 Were outcome assessors aware of the intervention received by study participants?	N	
	4.4 If Y/PY/NI to 4.3: Could assessment of the outcome have been influenced by knowledge of intervention received?	NA	
	4.5 If Y/PY/NI to 4.4: Is it likely that assessment of the outcome was influenced by knowledge of intervention received?	NA	
	**Risk of bias judgement**	**Low**	
**Bias in selection of the reported result**	5.1 Were the data that produced this result analysed in accordance with a pre-specified analysis plan that was finalized before unblinded outcome data were available for analysis?	Y	
	5.2 ... multiple eligible outcome measurements (e.g. scales, definitions, time points) within the outcome domain?	N	
	5.3 ... multiple eligible analyses of the data?	N	
	**Risk of bias judgement**	**Low**	

**Overall bias**	**Risk of bias judgement**	**Low**	

**Unique ID**	sahin2023	**Study ID**		**Assessor**	DANIEL
**Ref or Label**		**Aim**	assignment to intervention (the 'intention-to-treat' effect)		
**Experimental**		**Comparator**		**Source**	Journal article(s)
**Outcome**		**Results**		**Weight**	1

Domain	Signalling question	Response	Comments
**Bias arising from the randomization process**	1.1 Was the allocation sequence random?	NI	
	1.2 Was the allocation sequence concealed until participants were enrolled and assigned to interventions?	NI	
	1.3 Did baseline differences between intervention groups suggest a problem with the randomization process?	PN	
	**Risk of bias judgement**	**High**	Não há informações referentes a randomização, metodologia insuficiente.
**Bias due to deviations from intended interventions**	2.1.Were participants aware of their assigned intervention during the trial?	Y	
	2.2.Were carers and people delivering the interventions aware of participants' assigned intervention during the trial?	NI	
	2.3. If Y/PY/NI to 2.1 or 2.2: Were there deviations from the intended intervention that arose because of the experimental context?	N	
	2.4 If Y/PY to 2.3: Were these deviations likely to have affected the outcome?	NA	
	2.5. If Y/PY/NI to 2.4: Were these deviations from intended intervention balanced between groups?	NA	
	2.6 Was an appropriate analysis used to estimate the effect of assignment to intervention?	Y	
	2.7 If N/PN/NI to 2.6: Was there potential for a substantial impact (on the result) of the failure to analyse participants in the group to which they were randomized?	NA	
	**Risk of bias judgement**	**Some concerns**	Aqui indico que também faltam informações quanto ao cegamento dos pacientes/avaliadores do estudo. Poderiam
**Bias due to missing outcome data**	3.1 Were data for this outcome available for all, or nearly all, participants randomized?	Y	
	3.2 If N/PN/NI to 3.1: Is there evidence that result was not biased by missing outcome data?	NA	
	3.3 If N/PN to 3.2: Could missingness in the outcome depend on its true value?	NA	

data	3.4 If Y/PY/NI to 3.3: Is it likely that missingness in the outcome depended on its true value?	NA	
	**Risk of bias judgement**	**Low**	
**Bias in measurement of the outcome**	4.1 Was the method of measuring the outcome inappropriate?	N	
	4.2 Could measurement or ascertainment of the outcome have differed between intervention groups?	N	
	4.3 Were outcome assessors aware of the intervention received by study participants?	Y	
	4.4 If Y/PY/NI to 4.3: Could assessment of the outcome have been influenced by knowledge of intervention received?	Y	
	4.5 If Y/PY/NI to 4.4: Is it likely that assessment of the outcome was influenced by knowledge of intervention received?	N	
	**Risk of bias judgement**	**Some concerns**	
**Bias in selection of the reported result**	5.1 Were the data that produced this result analysed in accordance with a pre-specified analysis plan that was finalized before unblinded outcome data were available for analysis?	NI	
	5.2 ... multiple eligible outcome measurements (e.g. scales, definitions, time points) within the outcome domain?	N	
	5.3 ... multiple eligible analyses of the data?	N	
	**Risk of bias judgement**	**Some concerns**	
**Overall bias**	**Risk of bias judgement**	**High**	Pontos importantes na metodologia referentes sobre o processão de alocação e randomização ficaram faltando; Faltou clareza

**Unique ID**	sari2023	**Study ID**		**Assessor**	DANIEL
**Ref or Label**		**Aim**	assignment to intervention (the 'intention-to-treat' effect)		
**Experimental**		**Comparator**		**Source**	Journal article(s)
**Outcome**		**Results**		**Weight**	1

Domain	Signalling question	Response	Comments
**Bias arising from the randomization process**	1.1 Was the allocation sequence random?	Y	
	1.2 Was the allocation sequence concealed until participants were enrolled and assigned to interventions?	Y	
	1.3 Did baseline differences between intervention groups suggest a problem with the randomization process?	PY	

	**Risk of bias judgement**	**Some concerns**	
**Bias due to deviations from intended interventions**	2.1.Were participants aware of their assigned intervention during the trial?	N	
	2.2.Were carers and people delivering the interventions aware of participants' assigned intervention during the trial?	NI	
	2.3. If Y/PY/NI to 2.1 or 2.2: Were there deviations from the intended intervention that arose because of the experimental context?	N	
	2.4 If Y/PY to 2.3: Were these deviations likely to have affected the outcome?	NA	
	2.5. If Y/PY/NI to 2.4: Were these deviations from intended intervention balanced between groups?	NA	
	2.6 Was an appropriate analysis used to estimate the effect of assignment to intervention?	Y	
	2.7 If N/PN/NI to 2.6: Was there potential for a substantial impact (on the result) of the failure to analyse participants in the group to which they were randomized?	NA	
	**Risk of bias judgement**	**Low**	
**Bias due to missing outcome data**	3.1 Were data for this outcome available for all, or nearly all, participants randomized?	PY	
	3.2 If N/PN/NI to 3.1: Is there evidence that result was not biased by missing outcome data?	NA	
	3.3 If N/PN to 3.2: Could missingness in the outcome depend on its true value?	NA	
	3.4 If Y/PY/NI to 3.3: Is it likely that missingness in the outcome depended on its true value?	NA	
	**Risk of bias judgement**	**Some concerns**	
**Bias in measurement of the outcome**	4.1 Was the method of measuring the outcome inappropriate?	N	
	4.2 Could measurement or ascertainment of the outcome have differed between intervention groups?	PY	
	4.3 Were outcome assessors aware of the intervention received by study participants?	NA	
	4.4 If Y/PY/NI to 4.3: Could assessment of the outcome have been influenced by knowledge of intervention received?	NA	
	4.5 If Y/PY/NI to 4.4: Is it likely that assessment of the outcome was influenced by knowledge of intervention received?	NA	
	**Risk of bias judgement**	**High**	
**Bias in selection of the reported result**	5.1 Were the data that produced this result analysed in accordance with a pre-specified analysis plan that was finalized before unblinded outcome data were available for analysis?	NI	
	5.2 ... multiple eligible outcome measurements (e.g. scales, definitions, time points) within the outcome domain?	N	
	5.3 ... multiple eligible analyses of the data?	N	

	**Risk of bias judgement**	**Some concerns**	
**Overall bias**	**Risk of bias judgement**	**High**	

**Unique ID**	shouffoer2011	**Study ID**		**Assessor**	DANIEL
**Ref or Label**		**Aim**	assignment to intervention (the 'intention-to-treat' effect)		
**Experimental**		**Comparator**		**Source**	Journal article(s)
**Outcome**		**Results**		**Weight**	1

Domain	Signalling question	Response	Comments
**Bias arising from the randomization process**	1.1 Was the allocation sequence random?	Y	
	1.2 Was the allocation sequence concealed until participants were enrolled and assigned to interventions?	NI	
	1.3 Did baseline differences between intervention groups suggest a problem with the randomization process?	N	
	**Risk of bias judgement**	**Some concerns**	
**Bias due to deviations from intended interventions**	2.1.Were participants aware of their assigned intervention during the trial?	Y	
	2.2.Were carers and people delivering the interventions aware of participants' assigned intervention during the trial?	Y	
	2.3. If Y/PY/NI to 2.1 or 2.2: Were there deviations from the intended intervention that arose because of the experimental context?	N	
	2.4 If Y/PY to 2.3: Were these deviations likely to have affected the outcome?	NA	
	2.5. If Y/PY/NI to 2.4: Were these deviations from intended intervention balanced between groups?	NA	
	2.6 Was an appropriate analysis used to estimate the effect of assignment to intervention?	Y	
	2.7 If N/PN/NI to 2.6: Was there potential for a substantial impact (on the result) of the failure to analyse participants in the group to which they were randomized?	NA	
	**Risk of bias judgement**	**Low**	
**Bias due to**	3.1 Were data for this outcome available for all, or nearly all, participants randomized?	Y	
	3.2 If N/PN/NI to 3.1: Is there evidence that result was not biased by missing outcome data?	NA	

Bias due to missing outcome data	3.3 If N/PN to 3.2: Could missingness in the outcome depend on its true value?	NA	
	3.4 If Y/PY/NI to 3.3: Is it likely that missingness in the outcome depended on its true value?	NA	
	**Risk of bias judgement**	**Low**	
Bias in measurement of the outcome	4.1 Was the method of measuring the outcome inappropriate?	N	
	4.2 Could measurement or ascertainment of the outcome have differed between intervention groups?	N	
	4.3 Were outcome assessors aware of the intervention received by study participants?	N	
	4.4 If Y/PY/NI to 4.3: Could assessment of the outcome have been influenced by knowledge of intervention received?	NA	
	4.5 If Y/PY/NI to 4.4: Is it likely that assessment of the outcome was influenced by knowledge of intervention received?	NA	
	**Risk of bias judgement**	**Low**	
Bias in selection of the reported result	5.1 Were the data that produced this result analysed in accordance with a pre-specified analysis plan that was finalized before unblinded outcome data were available for analysis?	Y	
	5.2 ... multiple eligible outcome measurements (e.g. scales, definitions, time points) within the outcome domain?	N	
	5.3 ... multiple eligible analyses of the data?	N	
	**Risk of bias judgement**	**Low**	
**Overall bias**	**Risk of bias judgement**	**Some concerns**	

**Unique ID**	yakut2021	**Study ID**		**Assessor**	DANIEL
**Ref or Label**		**Aim**	assignment to intervention (the 'intention-to-treat' effect)		
**Experimental**		**Comparator**		**Source**	Journal article(s)
**Outcome**		**Results**		**Weight**	1

Domain	Signalling question	Response	Comments
Bias arising from the randomization	1.1 Was the allocation sequence random?	Y	
	1.2 Was the allocation sequence concealed until participants were enrolled and assigned to interventions?	Y	

**the randomization process**	1.3 Did baseline differences between intervention groups suggest a problem with the randomization process?	N	
	**Risk of bias judgement**	**Low**	
**Bias due to deviations from intended interventions**	2.1.Were participants aware of their assigned intervention during the trial?	Y	
	2.2.Were carers and people delivering the interventions aware of participants' assigned intervention during the trial?	Y	
	2.3. If Y/PY/NI to 2.1 or 2.2: Were there deviations from the intended intervention that arose because of the experimental context?	N	
	2.4 If Y/PY to 2.3: Were these deviations likely to have affected the outcome?	NA	
	2.5. If Y/PY/NI to 2.4: Were these deviations from intended intervention balanced between groups?	NA	
	2.6 Was an appropriate analysis used to estimate the effect of assignment to intervention?	Y	
	2.7 If N/PN/NI to 2.6: Was there potential for a substantial impact (on the result) of the failure to analyse participants in the group to which they were randomized?	NA	
	**Risk of bias judgement**	**Low**	
**Bias due to missing outcome data**	3.1 Were data for this outcome available for all, or nearly all, participants randomized?	Y	
	3.2 If N/PN/NI to 3.1: Is there evidence that result was not biased by missing outcome data?	NA	
	3.3 If N/PN to 3.2: Could missingness in the outcome depend on its true value?	NA	
	3.4 If Y/PY/NI to 3.3: Is it likely that missingness in the outcome depended on its true value?	NA	
	**Risk of bias judgement**	**Low**	
**Bias in measurement of the outcome**	4.1 Was the method of measuring the outcome inappropriate?	N	
	4.2 Could measurement or ascertainment of the outcome have differed between intervention groups?	N	
	4.3 Were outcome assessors aware of the intervention received by study participants?	Y	
	4.4 If Y/PY/NI to 4.3: Could assessment of the outcome have been influenced by knowledge of intervention received?	N	
	4.5 If Y/PY/NI to 4.4: Is it likely that assessment of the outcome was influenced by knowledge of intervention received?	NA	
	**Risk of bias judgement**	**Low**	
**Bias in selection of**	5.1 Were the data that produced this result analysed in accordance with a pre-specified analysis plan that was finalized before unblinded outcome data were available for analysis?	Y	
	5.2 ... multiple eligible outcome measurements (e.g. scales, definitions, time points) within the outcome domain?	N	

**the reported result**	5.3 ... multiple eligible analyses of the data?	N	
	**Risk of bias judgement**	**Low**	
**Overall bias**	**Risk of bias judgement**	**Some concerns**	

**Unique ID**	mitropoulos2020	**Study ID**		**Assessor**	DANIEL
**Ref or Label**		**Aim**	adhering to intervention (the 'per-protocol' effect)	**The effect of adhering to intervention…**	non-adherence to their assigned intervention by trial participants
**Experimental**		**Comparator**		**Source**	Journal article(s)
**Outcome**		**Results**		**Weight**	1

**Domain**	**Signalling question**	**Response**	**Comments**
**Bias arising from the randomization process**	1.1 Was the allocation sequence random?	Y	
	1.2 Was the allocation sequence concealed until participants were enrolled and assigned to interventions?	Y	
	1.3 Did baseline differences between intervention groups suggest a problem with the randomization process?	PN	
	**Risk of bias judgement**	**Some concerns**	
**Bias due to deviations from intended interventions**	2.1 Were participants aware of their assigned intervention during the trial?	Y	
	2.2 Were carers and people delivering the interventions aware of participants' assigned intervention during the trial?	Y	
	2.3. [If applicable:] If Y/PY/NI to 2.1 or 2.2: Were important non-protocol interventions balanced across intervention groups?	NA	
	2.4. [If applicable:] Were there failures in implementing the intervention that could have affected the outcome?	NA	
	2.5. [If applicable:] Was there non-adherence to the assigned intervention regimen that could have affected participants' outcomes?	N	
	2.6. If N/PN/NI to 2.3, or Y/PY/NI to 2.4 or 2.5: Was an appropriate analysis used to estimate the effect of adhering to the intervention?	NA	
	**Risk of bias judgement**	**Some concerns**	
**Bias due to missing outcome data**	3.1 Were data for this outcome available for all, or nearly all, participants randomized?	Y	
	3.2 If N/PN/NI to 3.1: Is there evidence that result was not biased by missing outcome data?	NA	
	3.3 If N/PN to 3.2: Could missingness in the outcome depend on its true value?	NA	

data	3.4 If Y/PY/NI to 3.3: Is it likely that missingness in the outcome depended on its true value?	NA	
	**Risk of bias judgement**	**Low**	
**Bias in measurement of the outcome**	4.1 Was the method of measuring the outcome inappropriate?	N	
	4.2 Could measurement or ascertainment of the outcome have differed between intervention groups?	N	
	4.3 Were outcome assessors aware of the intervention received by study participants?	NI	
	4.4 If Y/PY/NI to 4.3: Could assessment of the outcome have been influenced by knowledge of intervention received?	Y	
	4.5 If Y/PY/NI to 4.4: Is it likely that assessment of the outcome was influenced by knowledge of intervention received?	N	
	**Risk of bias judgement**	**Some concerns**	
**Bias in selection of the reported result**	5.1 Were the data that produced this result analysed in accordance with a pre-specified analysis plan that was finalized before unblinded outcome data were available for analysis?	Y	
	5.2 ... multiple eligible outcome measurements (e.g. scales, definitions, time points) within the outcome domain?	N	
	5.3 ... multiple eligible analyses of the data?	N	
	**Risk of bias judgement**	**Low**	
**Overall bias**	**Risk of bias judgement**	**Some concerns**	

**Unique ID**	piga2014	**Study ID**		**Assessor**	DANIEL
**Ref or Label**		**Aim**	adhering to intervention (the 'per-protocol' effect)	**The effect of adhering to intervention…**	non-adherence to their assigned intervention by trial participants
**Experimental**		**Comparator**		**Source**	Journal article(s)
**Outcome**		**Results**		**Weight**	

Domain	Signalling question	Response	Comments
**Bias arising from the randomization process**	1.1 Was the allocation sequence random?	Y	
	1.2 Was the allocation sequence concealed until participants were enrolled and assigned to interventions?	NI	
	1.3 Did baseline differences between intervention groups suggest a problem with the randomization process?	N	

	**Risk of bias judgement**	**Some concerns**	
**Bias due to deviations from intended interventions**	2.1 Were participants aware of their assigned intervention during the trial?	NI	
	2.2 Were carers and people delivering the interventions aware of participants' assigned intervention during the trial?	Y	
	2.3. [If applicable:] If Y/PY/NI to 2.1 or 2.2: Were important non-protocol interventions balanced across intervention groups?	NA	
	2.4. [If applicable:] Were there failures in implementing the intervention that could have affected the outcome?	NA	
	2.5. [If applicable:] Was there non-adherence to the assigned intervention regimen that could have affected participants' outcomes?	N	
	2.6. If N/PN/NI to 2.3, or Y/PY/NI to 2.4 or 2.5: Was an appropriate analysis used to estimate the effect of adhering to the intervention?	NA	
	**Risk of bias judgement**	**Low**	
**Bias due to missing outcome data**	3.1 Were data for this outcome available for all, or nearly all, participants randomized?	NI	
	3.2 If N/PN/NI to 3.1: Is there evidence that result was not biased by missing outcome data?	N	
	3.3 If N/PN to 3.2: Could missingness in the outcome depend on its true value?	N	
	3.4 If Y/PY/NI to 3.3: Is it likely that missingness in the outcome depended on its true value?	NA	
	**Risk of bias judgement**	**Low**	
**Bias in measurement of the outcome**	4.1 Was the method of measuring the outcome inappropriate?	N	
	4.2 Could measurement or ascertainment of the outcome have differed between intervention groups?	N	
	4.3 Were outcome assessors aware of the intervention received by study participants?	NI	
	4.4 If Y/PY/NI to 4.3: Could assessment of the outcome have been influenced by knowledge of intervention received?	Y	
	4.5 If Y/PY/NI to 4.4: Is it likely that assessment of the outcome was influenced by knowledge of intervention received?	N	
	**Risk of bias judgement**	**Some concerns**	
**Bias in selection of the reported result**	5.1 Were the data that produced this result analysed in accordance with a pre-specified analysis plan that was finalized before unblinded outcome data were available for analysis?	Y	
	5.2 ... multiple eligible outcome measurements (e.g. scales, definitions, time points) within the outcome domain?	N	
	5.3 ... multiple eligible analyses of the data?	N	
	**Risk of bias judgement**	**Low**	

**Overall bias**	**Risk of bias judgement**	**Some concerns**	

**Unique ID**	Filippetti, 2020	**Study ID**		**Assessor**	Santos, LP
**Ref or Label**		**Aim**	assignment to intervention (the 'intention-to-treat' effect)		
**Experimental**		**Comparator**		**Source**	Journal article(s)
**Outcome**		**Results**		**Weight**	1

Domain	Signalling question	Response	Comments
**Bias arising from the randomization process**	1.1 Was the allocation sequence random?	Y	
	1.2 Was the allocation sequence concealed until participants were enrolled and assigned to interventions?	NI	
	1.3 Did baseline differences between intervention groups suggest a problem with the randomization process?	N	
	**Risk of bias judgement**	**Some concerns**	
**Bias due to deviations from intended interventions**	2.1.Were participants aware of their assigned intervention during the trial?	Y	
	2.2.Were carers and people delivering the interventions aware of participants' assigned intervention during the trial?	Y	
	2.3. If Y/PY/NI to 2.1 or 2.2: Were there deviations from the intended intervention that arose because of the experimental context?	NI	
	2.4 If Y/PY to 2.3: Were these deviations likely to have affected the outcome?	NA	
	2.5. If Y/PY/NI to 2.4: Were these deviations from intended intervention balanced between groups?	NA	
	2.6 Was an appropriate analysis used to estimate the effect of assignment to intervention?	Y	
	2.7 If N/PN/NI to 2.6: Was there potential for a substantial impact (on the result) of the failure to analyse participants in the group to which they were randomized?	NA	
	**Risk of bias judgement**	**Some concerns**	
**Bias due to missing outcome data**	3.1 Were data for this outcome available for all, or nearly all, participants randomized?	Y	
	3.2 If N/PN/NI to 3.1: Is there evidence that result was not biased by missing outcome data?	NA	
	3.3 If N/PN to 3.2: Could missingness in the outcome depend on its true value?	NA	
	3.4 If Y/PY/NI to 3.3: Is it likely that missingness in the outcome depended on its true value?	NA	

	**Risk of bias judgement**	**Low**	
**Bias in measurement of the outcome**	4.1 Was the method of measuring the outcome inappropriate?	N	
	4.2 Could measurement or ascertainment of the outcome have differed between intervention groups?	N	
	4.3 Were outcome assessors aware of the intervention received by study participants?	N	
	4.4 If Y/PY/NI to 4.3: Could assessment of the outcome have been influenced by knowledge of intervention received?	NA	
	4.5 If Y/PY/NI to 4.4: Is it likely that assessment of the outcome was influenced by knowledge of intervention received?	NA	
	**Risk of bias judgement**	**Low**	
**Bias in selection of the reported result**	5.1 Were the data that produced this result analysed in accordance with a pre-specified analysis plan that was finalized before unblinded outcome data were available for analysis?	Y	
	5.2 ... multiple eligible outcome measurements (e.g. scales, definitions, time points) within the outcome domain?	N	
	5.3 ... multiple eligible analyses of the data?	N	
	**Risk of bias judgement**	**Low**	
**Overall bias**	**Risk of bias judgement**	**Some concerns**	

**Unique ID**	Gokcen, 2022	**Study ID**		**Assessor**	Santos, LP
**Ref or Label**		**Aim**	assignment to intervention (the 'intention-to-treat' effect)		
**Experimental**		**Comparator**		**Source**	Journal article(s)
**Outcome**		**Results**		**Weight**	1

**Domain**	**Signalling question**	**Response**	**Comments**
**Bias arising from the randomization process**	1.1 Was the allocation sequence random?	Y	
	1.2 Was the allocation sequence concealed until participants were enrolled and assigned to interventions?	Y	
	1.3 Did baseline differences between intervention groups suggest a problem with the randomization process?	N	
	**Risk of bias judgement**	**Low**	

**Bias due to deviations from intended interventions**	2.1.Were participants aware of their assigned intervention during the trial?	Y	
	2.2.Were carers and people delivering the interventions aware of participants' assigned intervention during the trial?	Y	
	2.3. If Y/PY/NI to 2.1 or 2.2: Were there deviations from the intended intervention that arose because of the experimental context?	N	
	2.4 If Y/PY to 2.3: Were these deviations likely to have affected the outcome?	NA	
	2.5. If Y/PY/NI to 2.4: Were these deviations from intended intervention balanced between groups?	NA	
	2.6 Was an appropriate analysis used to estimate the effect of assignment to intervention?	Y	
	2.7 If N/PN/NI to 2.6: Was there potential for a substantial impact (on the result) of the failure to analyse participants in the group to which they were randomized?	NA	
	**Risk of bias judgement**	**Low**	
**Bias due to missing outcome data**	3.1 Were data for this outcome available for all, or nearly all, participants randomized?	Y	
	3.2 If N/PN/NI to 3.1: Is there evidence that result was not biased by missing outcome data?	NA	
	3.3 If N/PN to 3.2: Could missingness in the outcome depend on its true value?	NA	
	3.4 If Y/PY/NI to 3.3: Is it likely that missingness in the outcome depended on its true value?	NA	
	**Risk of bias judgement**	**Low**	
**Bias in measurement of the outcome**	4.1 Was the method of measuring the outcome inappropriate?	PN	
	4.2 Could measurement or ascertainment of the outcome have differed between intervention groups?	N	
	4.3 Were outcome assessors aware of the intervention received by study participants?	NI	
	4.4 If Y/PY/NI to 4.3: Could assessment of the outcome have been influenced by knowledge of intervention received?	N	
	4.5 If Y/PY/NI to 4.4: Is it likely that assessment of the outcome was influenced by knowledge of intervention received?	NA	
	**Risk of bias judgement**	**Low**	
**Bias in selection of the reported result**	5.1 Were the data that produced this result analysed in accordance with a pre-specified analysis plan that was finalized before unblinded outcome data were available for analysis?	Y	
	5.2 ... multiple eligible outcome measurements (e.g. scales, definitions, time points) within the outcome domain?	N	
	5.3 ... multiple eligible analyses of the data?	N	
	**Risk of bias judgement**	**Low**	

Overall bias	Risk of bias judgement	Some concerns	

Unique ID	Roque, 2022	Study ID		Assessor	Santos, LP
Ref or Label		Aim	assignment to intervention (the 'intention-to-treat' effect)		
Experimental		Comparator		Source	Journal article(s)
Outcome		Results		Weight	1

Domain	Signalling question	Response	Comments
Bias arising from the randomization process	1.1 Was the allocation sequence random?	Y	
	1.2 Was the allocation sequence concealed until participants were enrolled and assigned to interventions?	Y	
	1.3 Did baseline differences between intervention groups suggest a problem with the randomization process?	N	
	**Risk of bias judgement**	**Low**	
Bias due to deviations from intended interventions	2.1.Were participants aware of their assigned intervention during the trial?	Y	
	2.2.Were carers and people delivering the interventions aware of participants' assigned intervention during the trial?	Y	
	2.3. If Y/PY/NI to 2.1 or 2.2: Were there deviations from the intended intervention that arose because of the experimental context?	N	
	2.4 If Y/PY to 2.3: Were these deviations likely to have affected the outcome?	NA	
	2.5. If Y/PY/NI to 2.4: Were these deviations from intended intervention balanced between groups?	NA	
	2.6 Was an appropriate analysis used to estimate the effect of assignment to intervention?	Y	
	2.7 If N/PN/NI to 2.6: Was there potential for a substantial impact (on the result) of the failure to analyse participants in the group to which they were randomized?	NA	
	**Risk of bias judgement**	**Low**	
Bias due to missing outcome data	3.1 Were data for this outcome available for all, or nearly all, participants randomized?	Y	
	3.2 If N/PN/NI to 3.1: Is there evidence that result was not biased by missing outcome data?	NA	
	3.3 If N/PN to 3.2: Could missingness in the outcome depend on its true value?	NA	

data	3.4 If Y/PY/NI to 3.3: Is it likely that missingness in the outcome depended on its true value?	NA	
	**Risk of bias judgement**	**Low**	
**Bias in measurement of the outcome**	4.1 Was the method of measuring the outcome inappropriate?	N	
	4.2 Could measurement or ascertainment of the outcome have differed between intervention groups?	N	
	4.3 Were outcome assessors aware of the intervention received by study participants?	N	
	4.4 If Y/PY/NI to 4.3: Could assessment of the outcome have been influenced by knowledge of intervention received?	NA	
	4.5 If Y/PY/NI to 4.4: Is it likely that assessment of the outcome was influenced by knowledge of intervention received?	NA	
	**Risk of bias judgement**	**Low**	
**Bias in selection of the reported result**	5.1 Were the data that produced this result analysed in accordance with a pre-specified analysis plan that was finalized before unblinded outcome data were available for analysis?	Y	
	5.2 ... multiple eligible outcome measurements (e.g. scales, definitions, time points) within the outcome domain?	N	
	5.3 ... multiple eligible analyses of the data?	N	
	**Risk of bias judgement**	**Low**	
**Overall bias**	**Risk of bias judgement**	**Low**	

**Unique ID**	Sahin, 2023	**Study ID**		**Assessor**	Santos, LP
**Ref or Label**		**Aim**	assignment to intervention (the 'intention-to-treat' effect)		
**Experimental**		**Comparator**		**Source**	Journal article(s)
**Outcome**		**Results**		**Weight**	1

Domain	Signalling question	Response	Comments
**Bias arising from the randomization process**	1.1 Was the allocation sequence random?	Y	
	1.2 Was the allocation sequence concealed until participants were enrolled and assigned to interventions?	NI	
	1.3 Did baseline differences between intervention groups suggest a problem with the randomization process?	PN	

	**Risk of bias judgement**	**Some concerns**	
**Bias due to deviations from intended interventions**	2.1.Were participants aware of their assigned intervention during the trial?	Y	
	2.2.Were carers and people delivering the interventions aware of participants' assigned intervention during the trial?	NI	
	2.3. If Y/PY/NI to 2.1 or 2.2: Were there deviations from the intended intervention that arose because of the experimental context?	N	
	2.4 If Y/PY to 2.3: Were these deviations likely to have affected the outcome?	NA	
	2.5. If Y/PY/NI to 2.4: Were these deviations from intended intervention balanced between groups?	NA	
	2.6 Was an appropriate analysis used to estimate the effect of assignment to intervention?	Y	
	2.7 If N/PN/NI to 2.6: Was there potential for a substantial impact (on the result) of the failure to analyse participants in the group to which they were randomized?	NA	
	**Risk of bias judgement**	**Some concerns**	
**Bias due to missing outcome data**	3.1 Were data for this outcome available for all, or nearly all, participants randomized?	Y	
	3.2 If N/PN/NI to 3.1: Is there evidence that result was not biased by missing outcome data?	NA	
	3.3 If N/PN to 3.2: Could missingness in the outcome depend on its true value?	NA	
	3.4 If Y/PY/NI to 3.3: Is it likely that missingness in the outcome depended on its true value?	NA	
	**Risk of bias judgement**	**Low**	
**Bias in measurement of the outcome**	4.1 Was the method of measuring the outcome inappropriate?	N	
	4.2 Could measurement or ascertainment of the outcome have differed between intervention groups?	N	
	4.3 Were outcome assessors aware of the intervention received by study participants?	Y	
	4.4 If Y/PY/NI to 4.3: Could assessment of the outcome have been influenced by knowledge of intervention received?	Y	
	4.5 If Y/PY/NI to 4.4: Is it likely that assessment of the outcome was influenced by knowledge of intervention received?	N	
	**Risk of bias judgement**	**Some concerns**	
**Bias in selection of the reported result**	5.1 Were the data that produced this result analysed in accordance with a pre-specified analysis plan that was finalized before unblinded outcome data were available for analysis?	NI	
	5.2 ... multiple eligible outcome measurements (e.g. scales, definitions, time points) within the outcome domain?	N	
	5.3 ... multiple eligible analyses of the data?	N	

	**Risk of bias judgement**	**Low**	
**Overall bias**	**Risk of bias judgement**	**High**	

**Unique ID**	Shouffoer, 2011	**Study ID**		**Assessor**	Santos, LP
**Ref or Label**		**Aim**	assignment to intervention (the 'intention-to-treat' effect)		
**Experimental**		**Comparator**		**Source**	Journal article(s)
**Outcome**		**Results**		**Weight**	1

Domain	Signalling question	Response	Comments
**Bias arising from the randomization process**	1.1 Was the allocation sequence random?	Y	
	1.2 Was the allocation sequence concealed until participants were enrolled and assigned to interventions?	NI	
	1.3 Did baseline differences between intervention groups suggest a problem with the randomization process?	N	
	**Risk of bias judgement**	**Some concerns**	
**Bias due to deviations from intended interventions**	2.1.Were participants aware of their assigned intervention during the trial?	Y	
	2.2.Were carers and people delivering the interventions aware of participants' assigned intervention during the trial?	Y	
	2.3. If Y/PY/NI to 2.1 or 2.2: Were there deviations from the intended intervention that arose because of the experimental context?	N	
	2.4 If Y/PY to 2.3: Were these deviations likely to have affected the outcome?	NA	
	2.5. If Y/PY/NI to 2.4: Were these deviations from intended intervention balanced between groups?	NA	
	2.6 Was an appropriate analysis used to estimate the effect of assignment to intervention?	Y	
	2.7 If N/PN/NI to 2.6: Was there potential for a substantial impact (on the result) of the failure to analyse participants in the group to which they were randomized?	NA	
	**Risk of bias judgement**	**Low**	
**Bias due to**	3.1 Were data for this outcome available for all, or nearly all, participants randomized?	Y	
	3.2 If N/PN/NI to 3.1: Is there evidence that result was not biased by missing outcome data?	NA	

Bias due to missing outcome data	3.3 If N/PN to 3.2: Could missingness in the outcome depend on its true value?	NA	
	3.4 If Y/PY/NI to 3.3: Is it likely that missingness in the outcome depended on its true value?	NA	
	**Risk of bias judgement**	**Low**	
**Bias in measurement of the outcome**	4.1 Was the method of measuring the outcome inappropriate?	N	
	4.2 Could measurement or ascertainment of the outcome have differed between intervention groups?	N	
	4.3 Were outcome assessors aware of the intervention received by study participants?	N	
	4.4 If Y/PY/NI to 4.3: Could assessment of the outcome have been influenced by knowledge of intervention received?	NA	
	4.5 If Y/PY/NI to 4.4: Is it likely that assessment of the outcome was influenced by knowledge of intervention received?	NA	
	**Risk of bias judgement**	**Low**	
**Bias in selection of the reported result**	5.1 Were the data that produced this result analysed in accordance with a pre-specified analysis plan that was finalized before unblinded outcome data were available for analysis?	Y	
	5.2 ... multiple eligible outcome measurements (e.g. scales, definitions, time points) within the outcome domain?	N	
	5.3 ... multiple eligible analyses of the data?	N	
	**Risk of bias judgement**	**Low**	
**Overall bias**	**Risk of bias judgement**	**Some concerns**	

**Unique ID**	Yakut, 2021	**Study ID**		**Assessor**	Santos, LP
**Ref or Label**		**Aim**	assignment to intervention (the 'intention-to-treat' effect)		
**Experimental**		**Comparator**		**Source**	Journal article(s)
**Outcome**		**Results**		**Weight**	1

Domain	Signalling question	Response	Comments
**Bias arising from the randomization**	1.1 Was the allocation sequence random?	Y	
	1.2 Was the allocation sequence concealed until participants were enrolled and assigned to interventions?	Y	

the randomization process	1.3 Did baseline differences between intervention groups suggest a problem with the randomization process?	N	
	**Risk of bias judgement**	**Low**	
**Bias due to deviations from intended interventions**	2.1.Were participants aware of their assigned intervention during the trial?	Y	
	2.2.Were carers and people delivering the interventions aware of participants' assigned intervention during the trial?	Y	
	2.3. If Y/PY/NI to 2.1 or 2.2: Were there deviations from the intended intervention that arose because of the experimental context?	N	
	2.4 If Y/PY to 2.3: Were these deviations likely to have affected the outcome?	NA	
	2.5. If Y/PY/NI to 2.4: Were these deviations from intended intervention balanced between groups?	NA	
	2.6 Was an appropriate analysis used to estimate the effect of assignment to intervention?	Y	
	2.7 If N/PN/NI to 2.6: Was there potential for a substantial impact (on the result) of the failure to analyse participants in the group to which they were randomized?	NA	
	**Risk of bias judgement**	**Low**	
**Bias due to missing outcome data**	3.1 Were data for this outcome available for all, or nearly all, participants randomized?	Y	
	3.2 If N/PN/NI to 3.1: Is there evidence that result was not biased by missing outcome data?	NA	
	3.3 If N/PN to 3.2: Could missingness in the outcome depend on its true value?	NA	
	3.4 If Y/PY/NI to 3.3: Is it likely that missingness in the outcome depended on its true value?	NA	
	**Risk of bias judgement**	**Low**	
**Bias in measurement of the outcome**	4.1 Was the method of measuring the outcome inappropriate?	N	
	4.2 Could measurement or ascertainment of the outcome have differed between intervention groups?	N	
	4.3 Were outcome assessors aware of the intervention received by study participants?	Y	
	4.4 If Y/PY/NI to 4.3: Could assessment of the outcome have been influenced by knowledge of intervention received?	N	
	4.5 If Y/PY/NI to 4.4: Is it likely that assessment of the outcome was influenced by knowledge of intervention received?	NA	
	**Risk of bias judgement**	**Low**	
**Bias in selection of**	5.1 Were the data that produced this result analysed in accordance with a pre-specified analysis plan that was finalized before unblinded outcome data were available for analysis?	Y	
	5.2 ... multiple eligible outcome measurements (e.g. scales, definitions, time points) within the outcome domain?	N	

the reported result	5.3 ... multiple eligible analyses of the data?	N	
	**Risk of bias judgement**	**Low**	
**Overall bias**	**Risk of bias judgement**	**Low**	

**Unique ID**	Horváth, 2017	**Study ID**		**Assessor**	Santos, LP
**Ref or Label**		**Aim**	assignment to intervention (the 'intention-to-treat' effect)		
**Experimental**		**Comparator**		**Source**	Journal article(s)
**Outcome**		**Results**		**Weight**	1

Domain	Signalling question	Response	Comments
**Bias arising from the randomization process**	1.1 Was the allocation sequence random?	N	
	1.2 Was the allocation sequence concealed until participants were enrolled and assigned to interventions?	NI	
	1.3 Did baseline differences between intervention groups suggest a problem with the randomization process?	N	
	**Risk of bias judgement**	**Some concerns**	
**Bias due to deviations from intended interventions**	2.1.Were participants aware of their assigned intervention during the trial?	PN	
	2.2.Were carers and people delivering the interventions aware of participants' assigned intervention during the trial?	N	
	2.3. If Y/PY/NI to 2.1 or 2.2: Were there deviations from the intended intervention that arose because of the experimental context?	NA	
	2.4 If Y/PY to 2.3: Were these deviations likely to have affected the outcome?	NA	
	2.5. If Y/PY/NI to 2.4: Were these deviations from intended intervention balanced between groups?	NA	
	2.6 Was an appropriate analysis used to estimate the effect of assignment to intervention?	PY	But the authors could have used ANOVA or GEE.
	2.7 If N/PN/NI to 2.6: Was there potential for a substantial impact (on the result) of the failure to analyse participants in the group to which they were randomized?	NA	

	**Risk of bias judgement**	**Low**	
**Bias due to missing outcome data**	3.1 Were data for this outcome available for all, or nearly all, participants randomized?	PY	
	3.2 If N/PN/NI to 3.1: Is there evidence that result was not biased by missing outcome data?	NA	
	3.3 If N/PN to 3.2: Could missingness in the outcome depend on its true value?	NA	
	3.4 If Y/PY/NI to 3.3: Is it likely that missingness in the outcome depended on its true value?	NA	
	**Risk of bias judgement**	**Low**	
**Bias in measurement of the outcome**	4.1 Was the method of measuring the outcome inappropriate?	N	
	4.2 Could measurement or ascertainment of the outcome have differed between intervention groups?	N	
	4.3 Were outcome assessors aware of the intervention received by study participants?	PN	
	4.4 If Y/PY/NI to 4.3: Could assessment of the outcome have been influenced by knowledge of intervention received?	NA	
	4.5 If Y/PY/NI to 4.4: Is it likely that assessment of the outcome was influenced by knowledge of intervention received?	NA	
	**Risk of bias judgement**	**Low**	
**Bias in selection of the reported result**	5.1 Were the data that produced this result analysed in accordance with a pre-specified analysis plan that was finalized before unblinded outcome data were available for analysis?	PN	Selecting patients in consecutive order for a specific protocol can send these analyses
	5.2 ... multiple eligible outcome measurements (e.g. scales, definitions, time points) within the outcome domain?	N	
	5.3 ... multiple eligible analyses of the data?	N	
	**Risk of bias judgement**	**Some concerns**	
**Overall bias**	**Risk of bias judgement**	**Some concerns**	

**Unique ID**	Sari, 2023	**Study ID**		**Assessor**	Santos, LP
**Ref or Label**		**Aim**	assignment to intervention (the 'intention-to-treat' effect)		
**Experimental**		**Comparator**		**Source**	Journal article(s)
**Outcome**		**Results**		**Weight**	1

Domain	Signalling question	Response	Comments
Bias arising from the randomization process	1.1 Was the allocation sequence random?	Y	
	1.2 Was the allocation sequence concealed until participants were enrolled and assigned to interventions?	Y	
	1.3 Did baseline differences between intervention groups suggest a problem with the randomization process?	PN	
	**Risk of bias judgement**	**Low**	
Bias due to deviations from intended interventions	2.1.Were participants aware of their assigned intervention during the trial?	N	
	2.2.Were carers and people delivering the interventions aware of participants' assigned intervention during the trial?	NI	
	2.3. If Y/PY/NI to 2.1 or 2.2: Were there deviations from the intended intervention that arose because of the experimental context?	PN	
	2.4 If Y/PY to 2.3: Were these deviations likely to have affected the outcome?	NA	
	2.5. If Y/PY/NI to 2.4: Were these deviations from intended intervention balanced between groups?	NA	
	2.6 Was an appropriate analysis used to estimate the effect of assignment to intervention?	PY	
	2.7 If N/PN/NI to 2.6: Was there potential for a substantial impact (on the result) of the failure to analyse participants in the group to which they were randomized?	NA	
	**Risk of bias judgement**	**Low**	
Bias due to missing outcome data	3.1 Were data for this outcome available for all, or nearly all, participants randomized?	PY	
	3.2 If N/PN/NI to 3.1: Is there evidence that result was not biased by missing outcome data?	NA	
	3.3 If N/PN to 3.2: Could missingness in the outcome depend on its true value?	NA	
	3.4 If Y/PY/NI to 3.3: Is it likely that missingness in the outcome depended on its true value?	NA	
	**Risk of bias judgement**	**Low**	
Bias in measurement of the outcome	4.1 Was the method of measuring the outcome inappropriate?	N	
	4.2 Could measurement or ascertainment of the outcome have differed between intervention groups?	N	
	4.3 Were outcome assessors aware of the intervention received by study participants?	NI	
	4.4 If Y/PY/NI to 4.3: Could assessment of the outcome have been influenced by knowledge of intervention received?	PY	
	4.5 If Y/PY/NI to 4.4: Is it likely that assessment of the outcome was influenced by knowledge of intervention received?	PN	

	**Risk of bias judgement**	**Some concerns**	
**Bias in selection of the reported result**	5.1 Were the data that produced this result analysed in accordance with a pre-specified analysis plan that was finalized before unblinded outcome data were available for analysis?	NI	
	5.2 ... multiple eligible outcome measurements (e.g. scales, definitions, time points) within the outcome domain?	PN	
	5.3 ... multiple eligible analyses of the data?	N	
	**Risk of bias judgement**	**Some concerns**	
**Overall bias**	**Risk of bias judgement**	**Some concerns**	

**Unique ID**	Piga, 2014	**Study ID**		**Assessor**	Santos, LP
**Ref or Label**		**Aim**	adhering to intervention (the 'per-protocol' effect)	**The effect of adhering to intervention…**	Non-adherence to their assigned intervention by trial participants
**Experimental**		**Comparator**		**Source**	Journal article(s)
**Outcome**		**Results**		**Weight**	1

Domain	Signalling question	Response	Comments
**Bias arising from the randomization process**	1.1 Was the allocation sequence random?	Y	
	1.2 Was the allocation sequence concealed until participants were enrolled and assigned to interventions?	NI	
	1.3 Did baseline differences between intervention groups suggest a problem with the randomization process?	PN	
	**Risk of bias judgement**	**Some concerns**	
**Bias due to deviations from intended interventions**	2.1 Were participants aware of their assigned intervention during the trial?	NI	
	2.2 Were carers and people delivering the interventions aware of participants' assigned intervention during the trial?	NI	
	2.3. [If applicable:] If Y/PY/NI to 2.1 or 2.2: Were important non-protocol interventions balanced across intervention groups?	NA	
	2.4. [If applicable:] Were there failures in implementing the intervention that could have affected the outcome?	NA	
	2.5. [If applicable:] Was there non-adherence to the assigned intervention regimen that could have affected participants' outcomes?	PY	
	2.6. If N/PN/NI to 2.3, or Y/PY/NI to 2.4 or 2.5: Was an appropriate analysis used to estimate the effect of adhering to the intervention?	PN	GEE analysis
	**Risk of bias judgement**	**High**	

**Bias due to missing outcome data**	3.1 Were data for this outcome available for all, or nearly all, participants randomized?	PY	Maybe a figure of adherence to controls was missing
	3.2 If N/PN/NI to 3.1: Is there evidence that result was not biased by missing outcome data?	NA	
	3.3 If N/PN to 3.2: Could missingness in the outcome depend on its true value?	NA	
	3.4 If Y/PY/NI to 3.3: Is it likely that missingness in the outcome depended on its true value?	NA	
	**Risk of bias judgement**	**Low**	
**Bias in measurement of the outcome**	4.1 Was the method of measuring the outcome inappropriate?	PN	
	4.2 Could measurement or ascertainment of the outcome have differed between intervention groups?	N	
	4.3 Were outcome assessors aware of the intervention received by study participants?	NI	
	4.4 If Y/PY/NI to 4.3: Could assessment of the outcome have been influenced by knowledge of intervention received?	Y	
	4.5 If Y/PY/NI to 4.4: Is it likely that assessment of the outcome was influenced by knowledge of intervention received?	PY	
	**Risk of bias judgement**	**High**	
**Bias in selection of the reported result**	5.1 Were the data that produced this result analysed in accordance with a pre-specified analysis plan that was finalized before unblinded outcome data were available for analysis?	NI	
	5.2 ... multiple eligible outcome measurements (e.g. scales, definitions, time points) within the outcome domain?	N	
	5.3 ... multiple eligible analyses of the data?	N	
	**Risk of bias judgement**	**Some concerns**	
**Overall bias**	**Risk of bias judgement**	**High**	

**Unique ID**	Mitropoulos, 2020	**Study ID**		**Assessor**	Santos, LP
**Ref or Label**		**Aim**	adhering to intervention (the 'per-protocol' effect)	**The effect of adhering to intervention...**	High adherence
**Experimental**		**Comparator**		**Source**	Journal article(s)

Outcome		Results		Weight	1

Domain	Signalling question	Response	Comments
Bias arising from the randomization process	1.1 Was the allocation sequence random?	Y	Patients were randomly allocated (block randomisation) between the exercise (n = 16) and control (n = 16) groups. The randomisation was performed by an independent statistician. The allocated group
	1.2 Was the allocation sequence concealed until participants were enrolled and assigned to interventions?	Y	
	1.3 Did baseline differences between intervention groups suggest a problem with the randomization process?	PY	Demographic data (mean ± SD) were shown without p values, and results may show statistical difference based on the mean per
	**Risk of bias judgement**	**Some concerns**	
Bias due to deviations from intended interventions	2.1 Were participants aware of their assigned intervention during the trial?	Y	
	2.2 Were carers and people delivering the interventions aware of participants' assigned intervention during the trial?	Y	
	2.3. [If applicable:] If Y/PY/NI to 2.1 or 2.2: Were important non-protocol interventions balanced across intervention groups?	PN	
	2.4. [If applicable:] Were there failures in implementing the intervention that could have affected the outcome?	NA	
	2.5. [If applicable:] Was there non-adherence to the assigned intervention regimen that could have affected participants' outcomes?	NA	
	2.6. If N/PN/NI to 2.3, or Y/PY/NI to 2.4 or 2.5: Was an appropriate analysis used to estimate the effect of adhering to the intervention?	N	The authors did not present information on statistical analysis
	**Risk of bias judgement**	**High**	
Bias due to missing outcome data	3.1 Were data for this outcome available for all, or nearly all, participants randomized?	Y	
	3.2 If N/PN/NI to 3.1: Is there evidence that result was not biased by missing outcome data?	NA	
	3.3 If N/PN to 3.2: Could missingness in the outcome depend on its true value?	NA	
	3.4 If Y/PY/NI to 3.3: Is it likely that missingness in the outcome depended on its true value?	NA	
	**Risk of bias judgement**	**Low**	
Bias in measurement of the outcome	4.1 Was the method of measuring the outcome inappropriate?	N	
	4.2 Could measurement or ascertainment of the outcome have differed between intervention groups?	N	
	4.3 Were outcome assessors aware of the intervention received by study participants?	NI	
	4.4 If Y/PY/NI to 4.3: Could assessment of the outcome have been influenced by knowledge of intervention received?	Y	It was not reported whether the test assessor was blinded.
	4.5 If Y/PY/NI to 4.4: Is it likely that assessment of the outcome was influenced by knowledge of intervention received?	Y	

	**Risk of bias judgement**	**High**	
**Bias in selection of the reported result**	5.1 Were the data that produced this result analysed in accordance with a pre-specified analysis plan that was finalized before unblinded outcome data were available for analysis?	Y	All data analysis was conducted at the end of data collection, using SPSS software (version 23, IBM SPSS, New York, USA).
	5.2 ... multiple eligible outcome measurements (e.g. scales, definitions, time points) within the outcome domain?	N	
	5.3 ... multiple eligible analyses of the data?	N	
	**Risk of bias judgement**	**Low**	
**Overall bias**	**Risk of bias judgement**	**High**	
