# Supplementary material for: Effects of interventions with resistance exercises on muscle strength, physical disability, and quality of life in systemic sclerosis patients: a systematic review with meta-analysis
Source: Adv Rheumatol. 2025 Jul 28;65:34. doi: 10.1186/s42358-025-00468-1 (PMC13488637; doi:10.1186/s42358-025-00468-1)
Supplement: Supplementary file 6 [file 42358_2025_468_MOESM6_ESM.pdf]

Autor(es):
Pergunta: Exercício resistido comparado a Controle (outros) para Esclerose sistêmica
Contexto:
Bibliografia:

Certainty assessment							№ de pacientes		Efeito		Certainty	Importância
№ dos estudos	Delineamento do estudo	Risco de viés	Inconsistência	Evidência indireta	Imprecisão	Outras considerações	Exercício resistido	Controle (outros)	Relativo (95% CI)	Absoluto (95% CI)		
**Muscle strength (seguimento: média 12 semanas; avaliado com: Handgrip test)**												
6	ensaios clínicos randomizados	grave[a]	não grave	não grave	grave[b]	nenhum	138	134	-	MD **2.78 Kg mais alto** (1.36 mais alto para 4.21 mais alto)	⊕⊕◯◯ Baixa	
**Physical disability (seguimento: média 12 semanas; avaliado com: HAQ-DI, SHAQ and HAQ)**												
5	ensaios clínicos randomizados	não grave	muito grave[c]	grave[d]	muito grave[b,e]	nenhum	115	104	-	SMD **0.5 SD menor** (0.78 menor para 0.23 menor)	⊕◯◯◯ Muito baixa	
**Quality of Life (seguimento: média 12 semanas; avaliado com: Mental component)**												
5	ensaios clínicos randomizados	não grave	não grave	grave[d]	muito grave[b,e]	nenhum	125	111	-	SMD **0.2 SD mais alto** (0.06 menor para 0.45 mais alto)	⊕◯◯◯ Muito baixa	
**Quality of Life (seguimento: média 12 semanas; avaliado com: Physical component)**												
5	ensaios clínicos randomizados	não grave	grave[f]	grave[d]	muito grave[b,e]	nenhum	115	104	-	SMD **0.42 SD mais alto** (0.04 mais alto para 0.81 mais alto)	⊕◯◯◯ Muito baixa	

CI: Confidence interval; **MD:** Mean difference; **SMD:** Standardised mean difference

Explanations

a. Of the six studies included in the meta-analysis, two (33%) had a high risk of bias.
b. Number of patients (<400)
c. i^2 = 64%
d. Different assessment instruments
e. Estimation of the effect on different benefit bands
f. I^2=48%

Author(s):
Question: Exercício resistido compared to controle for esclerose sistêmica
Setting:
Bibliography:

Certainty assessment							№ of patients		Effect		Certainty	Importance
№ of studies	Study design	Risk of bias	Inconsistency	Indirectness	Imprecision	Other considerations	Exercício resistido	controle	Relative (95% CI)	Absolute (95% CI)		
**Força Muscular (follow-up: mean 12 weeks; assessed with: Handgrip dynamometer; Scale from: 0 to 60)**												
6	randomised trials	not serious	not serious	not serious	serious[a]	none	138	134	-	MD **2.78 higher** (1.36 higher to 4.21 higher)	⨁⨁⨁◯ Moderate	IMPORTANT
**Capacidade funcional (follow-up: mean 12 weeks; assessed with: HAQ-DI; Scale from: 0 to 3)**												
5	randomised trials	not serious	serious[b]	not serious	serious[a]	none	115	104	-	SMD **0.5 SD lower** (0.78 lower to 0.23 lower)	⨁⨁◯◯ Low	IMPORTANT
**Qualidade de vida (mental) (follow-up: mean 12 weeks; assessed with: SF-36 e SF-12; Scale from: 0 to 100)**												
5	randomised trials	very serious[c]	not serious[b]	not serious	serious[a]	none	125	111	-	SMD **0.2 SD higher** (0.06 lower to 0.45 higher)	⨁◯◯◯ Very low	NOT IMPORTANT
**Qualidade de vida (físico) (follow-up: 12 weeks; assessed with: SF-36 e SF-12; Scale from: 0 to 100)**												
5	randomised trials	not serious	serious[b]	not serious	serious[a]	none	115	104	-	SMD **0.42 SD higher** (0.04 higher to 0.81 higher)	⨁⨁◯◯ Low	IMPORTANT

CI: confidence interval; **MD:** mean difference; **SMD:** standardised mean difference

Explanations

a. tamanho mínimo de informação não foi atingido (400 pacientes para desfechos quantitativos)
b. meta análise apresenta uma moderada heterogeneidade entre os estudos
c. intervalos de confiança que ultrapassam a linha central

Author(s):
Question: RE compared to Con for ES
Setting:
Bibliography:

Certainty assessment							№ of patients		Effect		Certainty	Importance
№ of studies	Study design	Risk of bias	Inconsistency	Indirectness	Imprecision	Other considerations	RE	Con	Relative (95% CI)	Absolute (95% CI)		
**Força (follow-up: mean 12 weeks; assessed with: Dinamometro preensão palmar)**												
6	randomised trials	serious[a]	not serious	not serious	serious[b]	none	138	134	-	MD **2.78 kg higher** (1.36 higher to 4.21 higher)	⨁⨁◯◯ Low	IMPORTANT
**Physical disability (follow-up: 12 weeks; assessed with: HAQ, HAQ-DI and SHAQ)**												
5	randomised trials	not serious	very serious[b,c]	serious[d]	very serious[b,e]	none	115	104	-	SMD **0.5 SD lower** (0.78 lower to 0.23 lower)	⨁◯◯◯ Very low	IMPORTANT
**Quality of life (M.C.) (follow-up: 12 weeks; assessed with: SF-36, SF-12)**												
5	randomised trials	very serious[f]	not serious	serious[d]	very serious[b,d]	none	125	111	-	SMD **0.2 SD higher** (0.6 higher to 0.45 higher)	⨁◯◯◯ Very low	IMPORTANT
**Quality of life (P.C.) (follow-up: 12 weeks; assessed with: SF-36, SF-12)**												
5	randomised trials	not serious	serious[g]	serious[d]	very serious[b,e]	none	115	104	-	SMD **0.42 SD higher** (0.04 higher to 0.81 higher)	⨁◯◯◯ Very low	IMPORTANT

CI: confidence interval; **MD:** mean difference; **SMD:** standardised mean difference

Explanations

a. Of the six studies included in the meta-analysis, two (33%) had a high risk of bias.
b. Number of patients (<400)
c. i2 = 64%
d. Different assessment methods
e. Estimation of the effect on different benefit bands
f. Confidence intervals
g. I2=48%
